# Colony growth and biofilm formation of *Aspergillus niger* under simulated microgravity

**DOI:** 10.3389/fmicb.2022.975763

**Published:** 2022-09-23

**Authors:** Marta Cortesão, Gudrun Holland, Tabea Schütze, Michael Laue, Ralf Moeller, Vera Meyer

**Affiliations:** ^1^German Aerospace Center (DLR), Institute of Aerospace Medicine, Radiation Biology Department, Aerospace Microbiology Research Group, Cologne, Germany; ^2^Chair of Applied and Molecular Microbiology, Institute of Biotechnology, Technische Universität Berlin, Berlin, Germany; ^3^Robert Koch Institute, Advanced Light and Electron Microscopy (ZBS 4), Berlin, Germany

**Keywords:** *Aspergillus niger*, simulated microgravity, biofilm, scanning electron microscopy, pigmentation, mycelium network, racA, fwnA

## Abstract

The biotechnology- and medicine-relevant fungus *Aspergillus niger* is a common colonizer of indoor habitats such as the International Space Station (ISS). Being able to colonize and biodegrade a wide range of surfaces, *A. niger* can ultimately impact human health and habitat safety. Surface contamination relies on two key-features of the fungal colony: the fungal spores, and the vegetative mycelium, also known as biofilm. Aboard the ISS, microorganisms and astronauts are shielded from extreme temperatures and radiation, but are inevitably affected by spaceflight microgravity. Knowing how microgravity affects *A. niger* colony growth, in particular regarding the vegetative mycelium (biofilm) and spore production, will help prevent and control fungal contaminations in indoor habitats on Earth and in space. Because fungal colonies grown on agar can be considered analogs for surface contamination, we investigated *A. niger* colony growth on agar in normal gravity (Ground) and simulated microgravity (SMG) conditions by fast-clinorotation. Three strains were included: a wild-type strain, a pigmentation mutant (Δ*fwnA*), and a hyperbranching mutant (Δ*racA*). Our study presents never before seen scanning electron microscopy (SEM) images of *A. niger* colonies that reveal a complex ultrastructure and biofilm architecture, and provide insights into fungal colony development, both on ground and in simulated microgravity. Results show that simulated microgravity affects colony growth in a strain-dependent manner, leading to thicker biofilms (vegetative mycelium) and increased spore production. We suggest that the Rho GTPase RacA might play a role in *A. niger*’s adaptation to simulated microgravity, as deletion of Δ*racA* leads to changes in biofilm thickness, spore production and total biomass. We also propose that FwnA-mediated melanin production plays a role in *A. niger*’s microgravity response, as Δ*fwnA* mutant colonies grown under SMG conditions showed increased colony area and spore production. Taken together, our study shows that simulated microgravity does not inhibit *A. niger* growth, but rather indicates a potential increase in surface-colonization. Further studies addressing fungal growth and surface contaminations in spaceflight should be conducted, not only to reduce the risk of negatively impacting human health and spacecraft material safety, but also to positively utilize fungal-based biotechnology to acquire needed resources *in situ*.

## Introduction

The filamentous fungus *Aspergillus niger* is valuably utilized in modern-day biotechnology and plays an important role in the transition to a bio-based circular economy (Cairns et al., 2018; Meyer et al., 2020). However, *A. niger* can also infect human lungs (Sugui et al., 2014), as well as colonize and biodegrade a wide range of surfaces, from surgical and construction materials, to water systems (Novikova, 2004; Klintworth et al., 1999; Yamaguchi et al., 2014). Indeed, *A. niger*’s ability to contaminate surfaces affects several indoor environments, from households, to hospitals, industrial facilities, airplane cabins and even the International Space Station (ISS) (Novikova et al., 2006, Di Pippo et al., 2018; National Research Council (Us) Committee on Air Quality in Passenger Cabins of Commercial Aircraft, 2002; Makimura et al., 2001; Vesper et al., 2008). The impact of fungal contaminations on human health and habitat safety highlights the need for effective prevention and decontamination strategies, which are only possible if we understand fungal surface-associated growth.

Surface contamination by *A. niger* relies on two key-features of the fungal colony: the fungal spores, which are the main reproductive structure of the fungus; and the vegetative mycelium, also known as biofilm, which interacts with, and biodegrades, the surface. Whether on agar, or on a kitchen wall, the first step for fungal colony development is spore adhesion to a surface. *A. niger* spores are highly resistant structures, partly due to the presence of pigments, such as melanin, on the outer layer of the spore’s cell wall. Melanin plays a major role in spore resistance to UV radiation (Cortesão et al., 2020a), and is also suggested to be involved in spore adhesion (Priegnitz et al., 2012). After adhering to a surface, spores then germinate and elongate into long cell threads named hyphae through a process known as polar growth. These hyphae branch frequently and ultimately create a complex interwoven network called vegetative mycelium, both on and in the substrate (Mela et al., 2020; Beauvais et al., 2014). Notably, the vegetative mycelium can be embedded in an extracellular matrix (ECM) (Sheppard and Howell, 2016; Chung and Brown, 2020) and can thus be considered a biofilm. In a fungal colony, it is the vegetative mycelium (biofilm) that interacts with the surface, promoting enzyme-driven substrate degradation as it grows and colonizes the surrounding environment (Gutarowska, 2010). Despite the importance of the fungal colony biofilm in surface contaminations, few studies have attempted to assess its complex morphology, cell network and ultrastructure (Krijgsheld et al., 2013). When nutrients become limited beneath the vegetative mycelium, a different type of hyphae, the aerial hyphae, start forming to explore the air space. Some of these aerial hyphae differentiate into conidiophores, with each conidiophore producing up to 10,000 spores. The conidiophores stand high above the vegetative mycelium, and use the natural air flow to release spores into the environment, which can then contaminate other surfaces (Gutarowska, 2010; Krijgsheld et al., 2013).

Control and prevention of fungal contaminations is challenging on Earth and is no different in a crewed spacecraft – the most extreme indoor-closed habitat (Zea et al., 2020; Cortesão et al., 2020b). Aboard the ISS, astronauts and microorganisms are shielded from extreme temperatures and radiation, but are inevitably affected by spaceflight microgravity. Spaceflight microgravity, of 10^–6^ g compared to 1g on the Earth’s surface, is known to induce changes in numerous cellular processes in microorganisms, from nutrient transport to virulence (Horneck et al., 2010; Rosenzweig et al., 2010; Eiermann et al., 2013; Hammond et al., 2013; Bizzarri et al., 2015; Taylor, 2015, Gilbert et al., 2020). However, whether spaceflight microgravity affects *A. niger* colony growth, and to which extent, is not yet understood (Zea et al., 2020; Cortesão et al., 2020b).

So far, only few studies have addressed the growth and adaptation of *A. niger* and related fungal species to microgravity aboard the ISS (Gomoiu et al., 2013, 2016; Romsdahl et al., 2018; Blachowicz et al., 2019). As experiments on the ISS are cost-intensive, difficult to implement, and extremely limited in throughput, Earth-based microgravity simulation methods are currently being used as alternative approaches to study adaptation phenomena of filamentous fungi to microgravity (Klaus, 2001; Hasenstein and van Loon, 2015). For example, previous studies with submerged liquid cultures of *A. niger* under low-shear modeled microgravity in the High Aspect Ratio Vessel (HARV), at a slow-rotation of 25 rpm, found no significant changes in spore germination, mycelium growth or cell wall integrity (Sathishkumar et al., 2014). Interestingly, further HARV studies at 30 rpm with the black fungus *Knufia chersonesos* found that low-shear modeled microgravity induced changes in the regulation of the DHN-melanin biosynthesis pathway (Tesei et al., 2021). Another study also reported that *A. niger* growth was not inhibited in a 3-D clinostat (Yamazaki et al., 2012). Also 2-D slow-clinorotation of solid media, at 20 rpm, has been used to generate simulated microgravity to study the growth of the food spoilage fungus *Aspergillus carbonarius*, revealing no changes in growth or colony appearance (Jiang et al., 2019). Recent studies with different microorganisms also used 2-D fast-clinorotation of solid media, at 60 rpm (Van Mulders et al., 2011; Ott et al., 2019; Garschagen et al., 2019), reporting that fast-clinorotation of green algae provided the closest simulation to that of real microgravity in the MAXUS sounding rocket (Krause et al., 2018).

Because *A. niger* colonies on an agar petri dish are grown in surface-associated, static and aerial conditions, they can be used as laboratory analogues for real-life fungal surface contaminations (Beauvais et al., 2007; Beauvais and Latgé, 2015; Lagree and Mitchell, 2017). Thus, in this study we investigated how microgravity affects *A. niger* colony growth and biofilm formation, in particular regarding the development and ultrastructure of the vegetative mycelium (biofilm), and regarding the production and integrity of its spores. For that we have grown colonies of *A. niger* in Ground conditions and in simulated microgravity (SMG) by fast-clinorotation of 60 rpm on a 2-D petri dish clinostat. Three different strains of *A. niger* were studied: an industrial wild-type strain (Bos et al., 1988), a strain defective in pigmentation (Δ*fwnA*) which is more susceptible to space radiation (Cortesão et al., 2020a; Jørgensen et al., 2011), and a hyperbranching mutant strain (Δ*racA*) of biotechnological interest (Kwon et al., 2011) that produces 20 % more hyphal tips than the wild-type, potentially influencing the complex hyphal network that is characteristic of a fungal colony biofilm. Our study presents unique scanning electron microscopy (SEM) images of colony cross fractures that show *A. niger*’s colony morphology and biofilm ultrastructure under Ground and simulated microgravity (SMG) conditions. We furthermore present the effect of simulated microgravity on *A. niger* colony area, biomass accumulation, spore production and spore integrity. Our study suggests the role of FwnA and RacA in *A. niger*’s adaptation to simulated microgravity, affecting colony growth, vegetative mycelium (biofilm) organization and spore production.

## Materials and methods

### Strains, media and culture conditions

Three *Aspergillus niger* strains were tested in this study and are summarized in Table 1: a fully pigmented wild-type strain (N402), a strain defective in pigment biosynthesis (MA93.1, Δ*fwnA*), and a strain defective in actin-controlled polar growth, with a hyperbranching phenotype (MA80.1, Δ*racA*). Spore suspensions were prepared from 3 days (for N402 and MA93.1) or 5 days-old (for MA80.1) colonies grown on complete media (CM) agar [55 mM glucose, 11 mM KH_2_PO_4_, 7 mM KCl, 178 nM H_3_BO_3_, 2 mM MgSO_4_, 76 nM ZnSO_4_, 70 mM NaNO_3_, 6.2 nM Na_2_MoO_4_, 18 nM FeSO_4_, 7.1 nM CoCl_2_, 6.4 nM CuSO_4_, 25 nM MnCl_2_, 174 nM EDTA, 15 g/L agar supplemented with 0.5 % (w/v) yeast extract and 0.1 % (w/v) casamino acids]. The spores were gently harvested with a sterile cotton swab, and suspended in 0.9 % sodium chloride (NaCl). The resulting spore suspensions were filtered through sterile Miracloth to remove hyphal fragments. Fresh spore suspensions, less than 2 weeks old, were used for all inoculations.

**TABLE 1:** *Aspergillus niger* strains used in this study.

Strain name	Relevant genotype	Characteristics	References
N402	Wild-type	Fully pigmented spores	Bos et al., 1988
MA93.1	Δ*fwnA*	Pigmentation mutant that produces fawn-colored spores due to the lack of the polyketide synthase FwnA	Jørgensen et al., 2011
MA80.1	Δ*racA*	Hyperbranching mutant due to the lack of the Rho GTPase RacA	Kwon et al., 2011

Standardized colony growth was achieved by inoculating 10 μL drop of a 10^6^ spores/ml spore suspension at the very center of the agar petri dishes, each with 20 mL of minimal medium (MM) agar [55 mM glucose, 11 mM KH_2_PO_4_, 7 mM KCl, 178 nM H_3_BO_3_, 2 mM MgSO_4_, 76 nM ZnSO_4_, 70 mM NaNO_3_, 6.2 nM Na_2_MoO_4_, 18 nM FeSO_4_, 7.1 nM CoCl_2_, 6.4 nM CuSO_4_, 25 nM MnCl_2_, 174 nM EDTA, 15 g/L agar], and incubating at 30°C for 3-5 days. Colonies were then grown under simulated microgravity using a clinostat (**see section 2.2**), or under normal gravitational conditions as a control (Ground). For growth assays 3-days old colonies were used, as their small radius limits the growth to the central region of the petri dish, where the quality of microgravity simulation in the clinostat is best. For SEM analysis investigating colony ultrastructure, 5-days old colonies were used, as they allow to study the ultrastructure of a fully mature and complex colony biofilm, and their larger radius allows easier handling during sample preparation, enabling sampling of distinct regions (center and edge) that represent different stages of maturation (older and younger). In contrast to the growth assays, the colonies used for SEM analysis were grown on top of a removable polycarbonate-filter (ø 47 mm, 0.4 μm pore size, hydrophilic, Merck Millipore, Darmstadt Germany). All filters were autoclaved in a glass petri dish (121°C, 20 min) and dried overnight at 22°C. Prior to inoculation, the filters were carefully placed on top of the agar plates using sterile tweezers. The filter allows to separate the colony from the underlying medium, which prevents the colonies from growing into the agar and enables whole colony retrieval for further preparation and scanning electron microscopy analysis (**see section 2.3**). There was no change in colony growth with and without the polycarbonate filter (data not shown).

### Clinostat cultivation

Clinostat cultivation to simulate microgravity has been earlier described for *Arabidopsis* seedlings, *Deinococcus radiodurans*, *Saccaromyces cerevisiae* (Van Mulders et al., 2011; Fengler et al., 2016, Ott et al., 2019) and for adherent mammalian cells (Eiermann et al., 2013). The clinostat simulates microgravity through continuous rotation around the horizontal axis (perpendicular to gravity), which averages the gravity vector close to zero, over time, for samples that are located directly in the center of the rotation axis, in this case the center of the petri dish (Herranz et al., 2013; Hasenstein and van Loon, 2015). This continuous rotation prevents particle sedimentation and exposes cells to a continuous free fall. It is important to note that the clinostat provides a functional simulation which is similar, but not identical, to that experienced in spaceflight microgravity. Besides, the quality of simulation is limited to the center of rotation, which in this case is the very center of the colonies. Thus, as the colony radius increases, acceleration and centrifugal forces increase.

Simulated microgravity at a given colony radius can be calculated as:


$$a=\omega^{2}\,r$$

Where a = centripetal acceleration (m s^–2^), ω = angular velocity (rad second^–1^) and r = colony radius (m). Here, angular velocity is calculated as:


$$\omega =2\,\pi \,{\mathrm{rpm60}}^{-1}$$

Where rpm = revolutions per minute (Anken, 2013), and 1 g = 9.81 m s^–1^.

**FIGURE 1:**
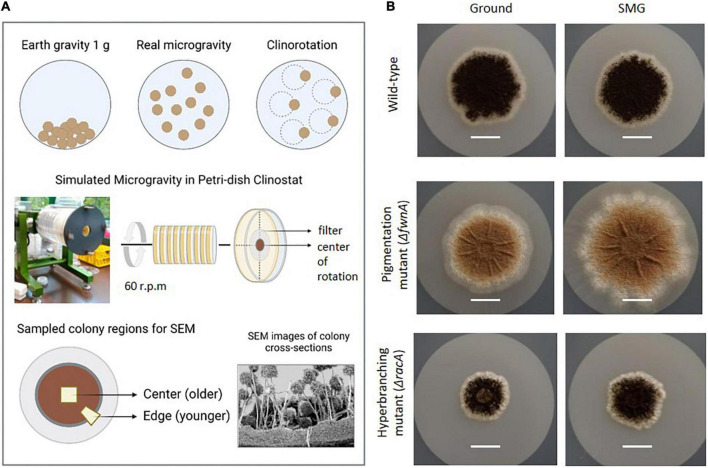
Simulation of microgravity in a petri dish clinostat. **(A)** Colonies were grown on a filter in petri dishes. These fit in the Clinostat, that simulates microgravity by continuously rotating the petri dishes (60 rpm). Here, the central region of the colony can achieve g-forces from 1.2 x 10^−2^ to 1.6 x 10^−2^ g, in a colony (r) radius between 0.3 – 0.4 cm. **(B)** Pictures of the colonies of the three tested strains comparing morphology and size b representative pictures of 5-days old colonies before SEM sample preparation (scale = 1 cm).

In this study, *A. niger* colonies were grown on minimal medium (MM) agar plates, and were rotated at fast-clinorotation speed of 60 rpm in a 2-D clinostat (Van Mulders et al., 2011; Eiermann et al., 2013; Krause et al., 2018; Garschagen et al., 2019). To analyze the simulated microgravity (SMG) effect of the tested growth parameters, 3 days-old colonies were used, as they have a small colony radius (<1 cm) and ensure exposure of the whole colony to simulated microgravity (4 x 10^–2^ g at colony radius = 1) (Anken, 2013). For SEM analysis, 5-days old colonies were used; however, it is important to note that to investigate simulated microgravity-induced morphological effects, only colony fragments at the center region were considered (*r* = 0.3 – 0.4 cm), given that region was exposed to high quality simulated microgravity, ranging between 1.2 x 10^–2^ to 1.6 x 10^–2^ g (Anken, 2013; Figure 1).

### Scanning electron microscopy

Colonies grown on filters were chemically fixed and freeze-fractured as previously described by Fuchs et al., 2018, with few modifications explained below. This method generates regular fractures perpendicular to the colony surface (cross fractures) through selected large areas of the colony which allows to study both, macroscopic architecture and ultrastructural detail, such as extracellular matrix or conidiophore heads.

Each colony-carrying filter was carefully transferred from the agar plate into a small petri dish (ø 5 cm) using sterile forceps and fixed by submerging the colonies in a mixture of 2.5 % glutaraldehyde and 1 % para-formaldehyde in 0.05 M HEPES buffer, for 12h at room temperature. Post-fixation was done with 1% osmium tetroxide in water before samples were dehydrated in ethanol (see Fuchs et al., 2018). During dehydration, colonies usually separated from the filter (without leaving any visible residual colony material on the filter) and some spores were lost. However, the overall integrity of the colony was preserved. To prepare freezing and fracturing, two samples (∼ 5 x 3 mm), one from center and one from the edge region, were extracted from each colony (in 100 % ethanol) by using a razor blade. Samples were frozen in liquid nitrogen and fractured according to Fuchs et al., 2018. However, fracturing of samples from the hyperbranching mutant was not successful using the foreceps-method, most probably because of the thicker vegetative mycelium in comparison to the other strains. Thus, we fractured samples from the hyperbranching mutant by using a cooled razor blade. After fracturing, sample fragments were thawed in 100 % ethanol and dried by critical point drying using an automated device (CPD 300, Leica, Wetzlar, Germany). Dried samples were mounted with fractures aligned upwards (90° angle) and sputter-coated with 15 nm gold/palladium (Sputter coater E5100, Polaron/Quorum Technologies, United Kingdom). Electron microscopy of colony fractures was carried out with a field-emission scanning electron microscope (Gemini 1530, Zeiss, Oberkochen, Germany) operated at 3 kV and a working distance of 5 mm. Signals from the in-lens secondary electron detector were used to investigate the samples with topography contrast.

SEM image evaluation was done with the iTEM software (Version 5.2; EMSIS, Germany), and with Digimizer image analysis software (MedCalc Software Ltd., Belgium). Thickness of the vegetative mycelium and height of the conidiophores were determined for the central regions of the colony (at least three measurements per image) of each strain, and each biological triplicate (*n* ≥ 9). Thickness of each vegetative mycelium layer of the Δ*racA* strain was determined by four measurements of the central regions per image of each biological replicate (*n* = 12). Measurements in folded regions of the Δ*racA* strain (Supplementary Figure 3) were not included in the mycelium thickness analysis, to allow for comparison between strains.

### Transmission electron microscopy

Small parts (a few millimeters) of colonies cultivated under normal gravitational conditions were extracted with a scalpel and sliced in thin sections by using a razor blade. The thin slabs were transferred to the shallow depth (300 μm) of an aluminum platelet (3 mm diameter) filled with 1-hexadecene, covered with a flat aluminum platelet and fixed by high-pressure freezing (HPF Compact 01, Engineering Office M. Wohlwend GmbH, Switzerland). Frozen samples were freeze-substituted in 0.2 % osmium tetroxide, 0.01 % uranyl acetate, 5 % H_2_O in acetone using an automated freeze-substitution device (AFS, Leica Microsystems, Germany) for 3 days and embedded in epon resin. Thin sections (60-80 nm) were produced with an ultramicrotome (UC7, Leica Microsystems, Germany) and contrasted with uranyl acetate and lead citrate. Electron microscopy was performed with a TEM (Tecnai Spirit, Thermo Fisher, The Netherlands) operated at 120 kV. Images were recorded with a CCD camera (Megaview III, EMSIS, Germany) (*n* = 2).

### Evaluation of colony growth: Area, biomass and spore yield

Colony area was determined by taking high resolution photographs of the colonies of each biological replicate (*n* = 3), using a camera (Sony α-500 APS-C) with a macro-objective (E 3.5/30), that was kept at constant height via a fixed tripod. Photographs were then transferred to Fiji/Image J software. Here, colony area was calculated by setting the scale and adjusting color and threshold, to account for the entirety of each colony (including submerged edges of the mycelium, which were able to be photographed through the agar). Colony biomass was determined via dried biomass where each colony-carrying filter, of each biological replicate (*n* = 3), was recovered and placed inside a pre-weighted aluminum paper. The weight of the aluminum with a colony was measured before (wet weight = ww) and after desiccation (dry weight = dw) for 24 h at 60 °C (at this point weight was constant). Weight was measured with a high precision analytical scale (Sartorius). Dry biomass of each colony was calculated as:


Colony⁢biomass=dry⁢weight⁢(dw)-wet⁢weight⁢(ww)

To determine the number of spores produced, spores were harvested from single colonies, of each biological replicate (*n* = 3), after 3 days on MM-agar plates without filter, by flooding the petri dish with 5 mL of 0.9 % sodium chloride, gently scraping the spores with a sterile cotton swab, and filtering the solution through sterile miracloth to filter out hyphal fragments. Spores were counted using a cell counting chamber (improved Neubauer) by light microscopy.

### Evaluation of spore integrity: Hydrophobicity, metabolic activity, and germination rate

To compare the relative hydrophobicity profiles of spores from tested *A. niger* strains, the Microbial Adhesion to Hydrocarbons (MATH) assay was adapted from Shuster et al., 2019 and Faille et al., 2014. Spores isolated from colonies of each biological replicate (*n* = 3) were suspended in 0.9 % sodium chloride and exposed to hexadecane, an apolar solvent, that allows spores to settle in either the aqueous or the organic phase, depended on which more strongly interacts with the spore surface. This semi-quantitative approach provides evidence of the surface hydrophobicity of the spores via their interactive properties. For that, 2.5 ml of spore suspension (1 x 10^6^ spores/ml) were added to glass tubes and 5 ml hexadecane were slowly added on top. The tubes were then vortexed for 2 min, followed by a 15 min settling phase, after which 1 ml sample of the suspension was taken carefully from the bottom of the tube (through the hexadecane layer). Measurements of spore suspensions at OD_600nm_ were taken before (N_0_) and after contact with hexadecane (N). Hydrophobicity was calculated as:


$$\left(\frac{\text{N}}{\mathrm{N0}}\,x\,100\right)-100$$

which determines the percentage of spores in the hydrocarbon layer, i.e., the higher the percentage of spores in the hydrocarbon layer, the higher the hydrophobicity. This experiment was performed with three technical replicates per tested strain (*n* = 3).

Spore germination rate was determined by inoculating three drops of 3 μL, each containing 10^6^ spores/ml, on MM-agar plates supplemented with 0.003% yeast extract, for each strain (*n* = 3). After incubation at 22°C for 22 h, a total of 200 spores and germlings were counted per replicate using light microscopy. Germination rate was calculated as the ratio between germinated (G) and non-germinated spores (NG), as follows:


$$\frac{\mathrm{germinated}\,\left(G\right)}{\mathrm{non}-\mathrm{germinated}\,\left(\mathrm{NG}\right)}$$

Metabolic activity of spores was measured in a 96-well plate, via detection of color changes during the metabolization (reduction) of resazurin (blue) into resorufin (pink), as the spores germinate and outgrow. For that each well contained 195 μl of minimal media (MM), 75 μL of spore suspension (10^6^ spores/ml), 30 μL of Alamar Blue (Sigma), in a total volume of 300 μL. Samples were then incubated up to 48 h at 30°C in a microplate reader (Infinite M200 PRO, Tecan) (*n* = 6), where OD_570 nm_ and OD_600 nm_ were measured every 30 min. The percentage of reduced resazurin reagent was calculated according to the standard protocol obtained from Thermo Fisher™.

### Data analysis

Data were plotted as means and error bars calculated as standard error. Graphs were plotted with Sigma Plot.14. Statistical analysis was done with Student’s t-test, using mean and standard-error between Ground and SMG conditions, or between a mutant strain and the wild-type. A one-tailed p-value of *p* ≤ 0.05 was considered as significant.

## Results

In this study, we investigated the effect of simulated microgravity in colony growth and biofilm ultrastructure of the industrially and medically relevant filamentous fungus *Aspergillus niger*, that is also a common colonizer of indoor environments such as the ISS. For that, biological triplicates of a fully-pigmented wild-type strain, a pigmentation mutant strain (Δ*fwnA*) and a hyperbranching mutant strain (Δ*racA*) were grown on agar under normal Earth gravity (Ground) and under simulated microgravity (SMG) conditions, in a fast-rotating petri dish clinostat (Figure 1). Results provide insights into *A. niger* colony development, and present never-before seen SEM images of colony cross fractures, revealing the complex morphology of *A. niger* vegetative mycelium (or biofilm). Results also inform on SMG-induced changes in colony morphology, i.e., area, dried biomass, biofilm thickness and conidiophore height; as well as changes in spore integrity, i.e., metabolic activity, spore hydrophobicity and germination rate.

### Colony growth of *Aspergillus niger* strains under normal gravitational conditions

**FIGURE 2:**
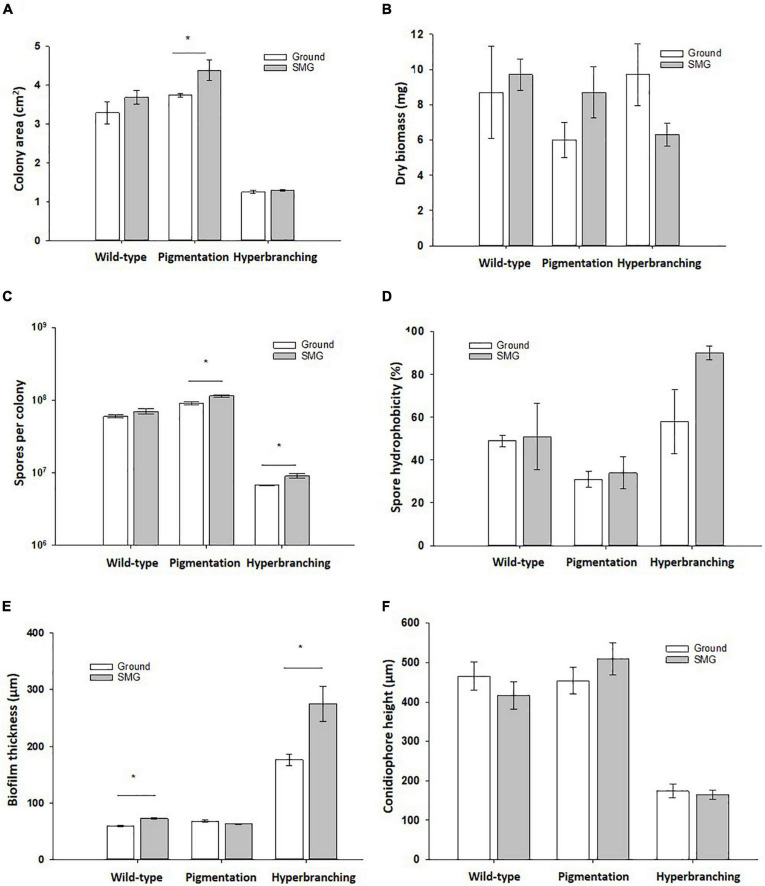
Effect of simulated microgravity in colony growth of *A. niger* strains. **(A)** Colony area, where lack of pigmentation leads to increased colony area in SMG. **(B)** Dry biomass. **(C)** Spore production, where lack of pigmentation leads to increased spore production in SMG. **(D)** Spore hydrophobicity, where there were no significant changes under SMG conditions. **(E)** Biofilm thickness, where wild-type and hyperbranching mutant form significantly thicker biofilms under simulated microgravity, and the hyperbranching mutant forms 3 x thicker biofilms than the wild-type, regardless of the gravitational regime. **(F)** Conidiophore height, where conidiophores of the hyperbranching mutant strain reach a significantly lower height than the wild-type. Data shown as Mean + SE, *p* ≤ 0.05 was considered significant (*).

When grown in normal gravitational conditions, 3-days old wild-type colonies reached an average area of 3.3 cm^2^, with each colony accumulating an average total of 8.7 mg of biomass and producing on average 6 x 10^7^ spores (Figure 2 and Table 2). In comparison, the pigmentation mutant, lacking the polyketide synthase FwnA (Δ*fwnA*) produced slightly larger colonies than the wild-type (on average 3.7 cm^2^) that produced significantly more spores (9 x 10^7^ spores, *p* = 0.04), but accumulated 30% less biomass (6 mg) than wild-type colonies. In contrast, colonies of the hyperbranching mutant, lacking the RhoGTPase RacA (Δ*racA*) were significantly smaller than wild-type colonies (on average 1.2 cm^2^, *p* = 0.02), but accumulated a similar total biomass (9.7 mg) due to its hyperbranching phenotype, as it was previously described (Kwon *et al.*, 2011). A deletion of Δ*racA* also resulted in significantly less spores than the wild-type (an average of 6.8 x 10^6^ spores per colony, *p* < 0.01) (Figure 2 and Table 2).

**TABLE 2:** Effect of simulated microgravity in *A. niger* colony biofilms.

Parameter	Strain	Ground	SMG	≠	p-value
Colony area (cm^2^)					
	Wild-type	3.3 ± 0.3	3.7 ± 0.2	↑	0.3
	Pigmentation	3.7 ± 0.2	4.4 ± 0.3	↑	0.07*
	Hyperbranching	1.2 ± 0.0	1.3 ± 0.0	↑	0.3
Dried biomass (mg)					
	Wild-type	8.7 ± 2.6	9,7 ± 0.9	=	0.7
	Pigmentation	6.0 ± 1.0	8.7 ± 1.5	↑	0.2
	Hyperbranching	9.7 ± 1.8	6.3 ± 0.7	↓	0.1
Spores per colony					
	Wild-type	6.0 x 10^7^ ± 2.4 x 10^6^	7.0 x 10^7^ ± 6.2 x 10^6^	↑	0.2
	Pigmentation	9.0 x 10^7^ ± 4.5 x 10^6^	1.1 x 10^8^ ± 5.4 x 10^6^	↑	0.03*
	Hyperbranching	6.8 x 10^6^ ± 1.3 x 10^5^	9.1 x 10^6^ ± 7.1 x 10^5^	↑	0.03*
Spore metabolic max (%)					
	Wild-type	17 ± 0.4	15 ± .09	↓	0.1
	Pigmentation	16 ± 1.1	17 ± 0.3	=	0.7
	Hyperbranching	19 ± 0.6	19 ± 1.1	=	0.8
Spore metabolic max (h)					
	Wild-type	25 ± 0.4	24 ± 0.4	↑	0.15
	Pigmentation	26 ± 0.3	26 ± 0.4	=	1
	Hyperbranching	24 ± 0.6	23 ± 0.4	↓	0.2
Spore germination (G/NG)					
	Wild-type	10 ± 0.4	5.6 ± 1	**↓**	0.02*
	Pigmentation	15 ± 1.1	15 ± 2.4	=	0.9
Spore hydrophobicity (%)					
	Wild-type	49 ± 3	51 ± 15	=	0.9
	Pigmentation	31 ± 4	34 ± 7	=	0.7
	Hyperbranching	58 ± 15	90 ± 3	↑	0.1
Biofilm thickness in colony center (μm)					
	Wild-type	59 ± 1	72 ± 1	↑	0.001*
	Pigmentation	67 ± 2	63 ± 1	↓	0.1
	Hyperbranching	176 ± 10	275 ± 31	↑	0.04*
Conidiophore height (μm)					
	Wild-type	466 ± 35	417 ± 35	↓	0.4
	Pigmentation	454 ± 34	510 ± 41	↑	0.4
	Hyperbranching	174 ± 17	165 ± 11	=	0.7

Three strains were tested: wild-type, pigmentation (Δ*fwnA*) and hyperbranching (Δ*racA*) mutant strains. *Statistical significance with Students t-test where one-tailed p ≤ 0.05.

**FIGURE 3:**
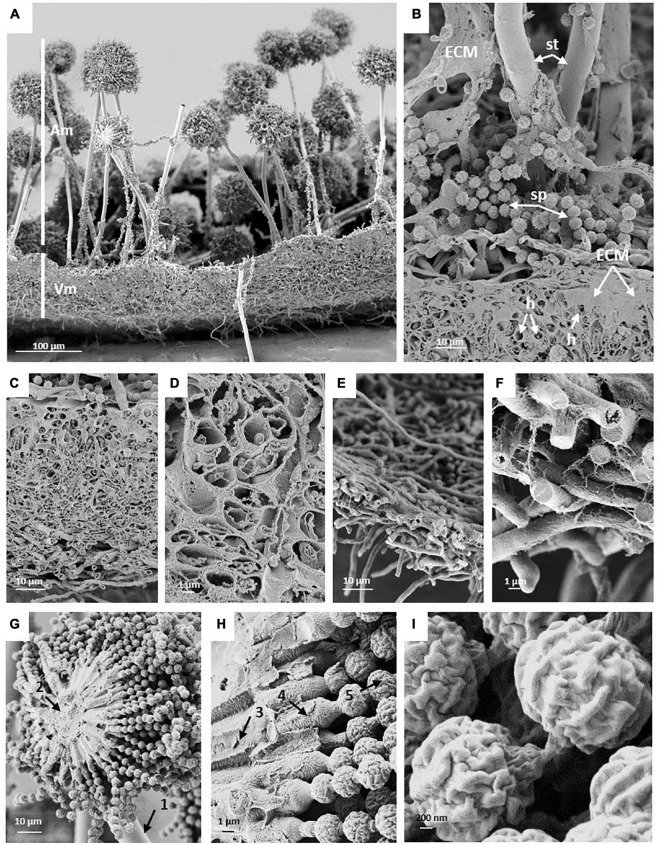
SEM of a cross fracture through an *A. niger* wild-type colony which was grown under normal gravitational conditions demonstrates typical structural elements. **(A)** Vegetative (Vm) and aerial mycelia of the center region with conidiophores (Am). **(B)** Close-up view of the interface of aerial and vegetative mycelium showing extracellular matrix (ECM), the stalk (st), loose spores (sp) and vegetative hyphae (h). **(C)** ECM covered vegetative mycelium (biofilm) in the center (oldest) region of the colony, a 5-days-old mature colony biofilm. **(D)** Hollow, almost empty appearing hyphae in the center region. **(E,F)** Hyphae at the edge of the colony (youngest region) are not embedded in a thick layer of ECM and appear filled **(F)**. **(G,H)** Cross fractures of *Aspergillus niger* conidiophore revealing (1) stalk; (2) vesicle; (3) primary sterigmata = metulae; (4) secondary sterigmata = phialide; and (5) conidia (spore) chains. **(I)** SEM of spores showing the cell wall with ridges.

**FIGURE 4:**
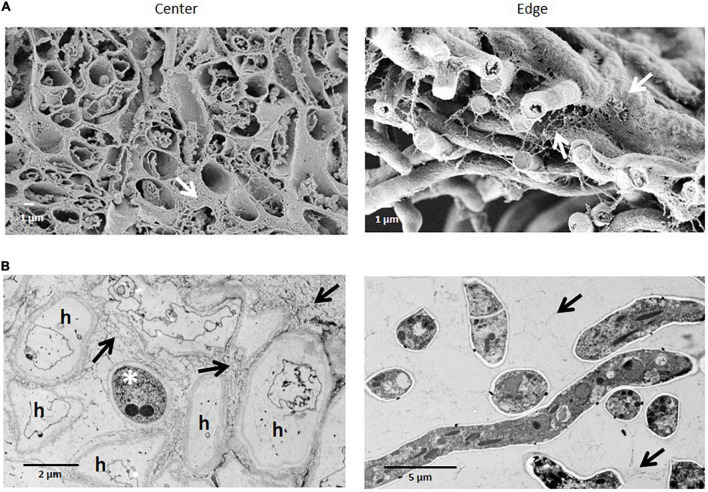
Comparison of the ultrastructural of *A. niger* hyphae in colony center and edge regions after chemical or high-pressure cryo-fixation. **(A)** SEM of chemically fixed samples. Cross fractures through the colony show almost empty, largely extracted hyphae in the center of a colony, and filled hyphae at the edge region of a colony. **(B)** TEM of ultrathin sections of high-pressure frozen samples show inactive hyphae (h) (except one asterisk), in the center of the colony, while at the edge hyphae reveal a dense, intact cytoplasm with all typical organelles. Arrows indicate the extracellular matrix formed.

SEM images of colony cross fractures from the wild-type strain revealed morphological differences among the different regions of the colony under normal gravitational conditions (Figure 3). Cross fractures of the youngest part of the colony (edge region) were populated with foraging hyphae filled with cytoplasm, connected in a loose network. In contrast, hyphae from the oldest part of the colony (central region) were devoid of cytoplasm, with only a few granular remnants likely left due to autophagic processes. This data was confirmed by transmission electron microscopy (TEM), for which we used another fixation method (high-pressure freezing instead of chemical fixation) in order to exclude the possibility that chemical fixation of SEM samples could have unintentionally altered hyphal morphologies (Figures 3C–F, 4). Indeed, autophagy in *Aspergillus* is known to be related with aging, is integral to nutrient and organelles recycling and is thought to facilitate growth of aerial hyphae and conidiophores (Kikuma et al., 2007; Nitsche et al., 2013; Lingo et al., 2021).

**FIGURE 5:**
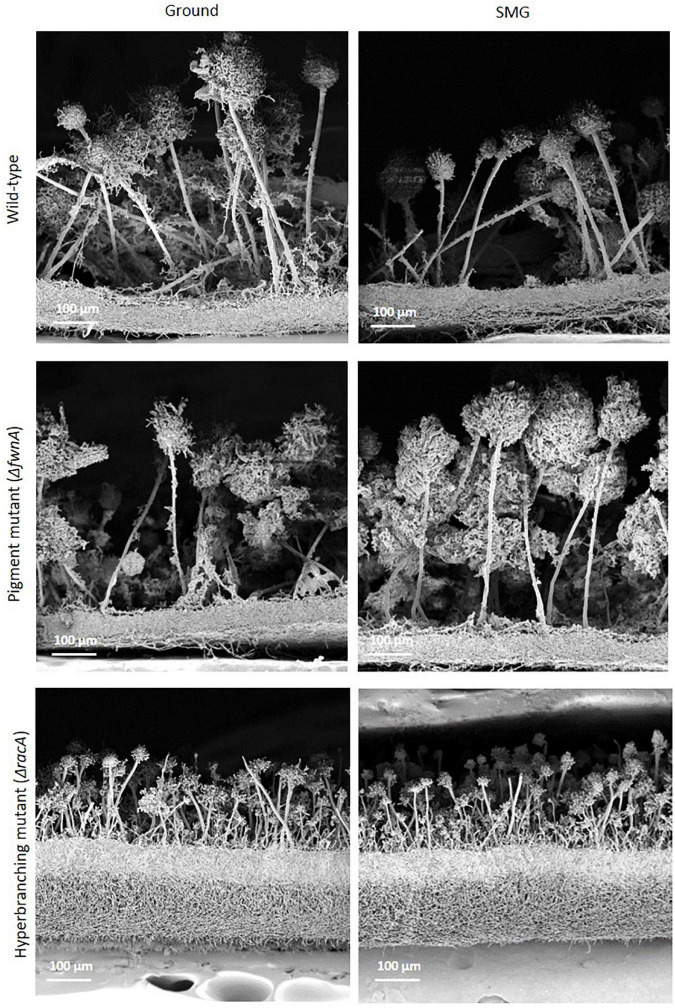
SEM of cross fractures through the central region of colonies of the three different strains of *A. niger* cultivated either under Ground or SMG conditions. While the morphology of vegetative (biofilm) and aerial mycelia of wild-type and pigmented mutant appear almost identical at this resolution (see Figure 6 for differences regarding the conidiophore heads), the vegetative mycelium of the hyperbranching mutant is much thicker and the conidiophores are shorter with smaller heads than in the two other strains. Differences between the two gravity conditions are not readily visible (but see Figure 2 and text).

**FIGURE 6:**
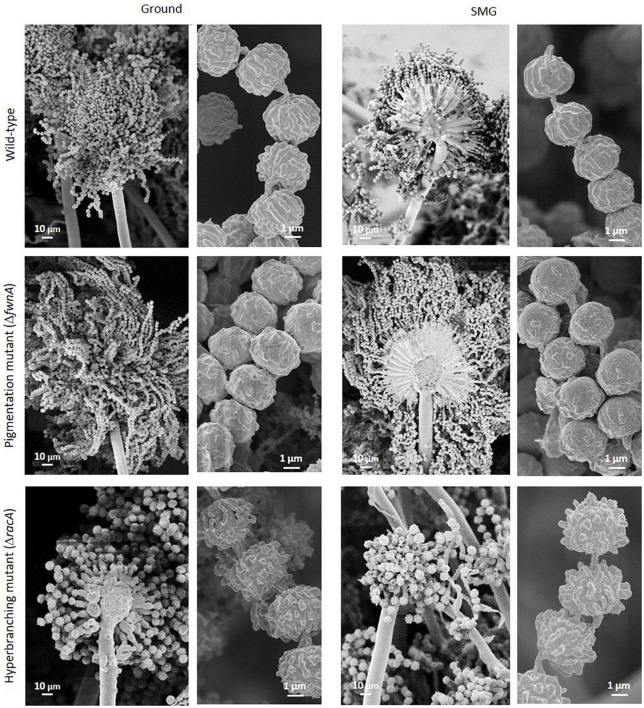
SEM of conidiophores and spore chains from colonies cultivated either under Ground or SMG conditions. Spore chains of the pigmentation mutant are longer and spore chains of hyperbranching mutant are shorter than the spore chains of the wild-type.

**FIGURE 7:**
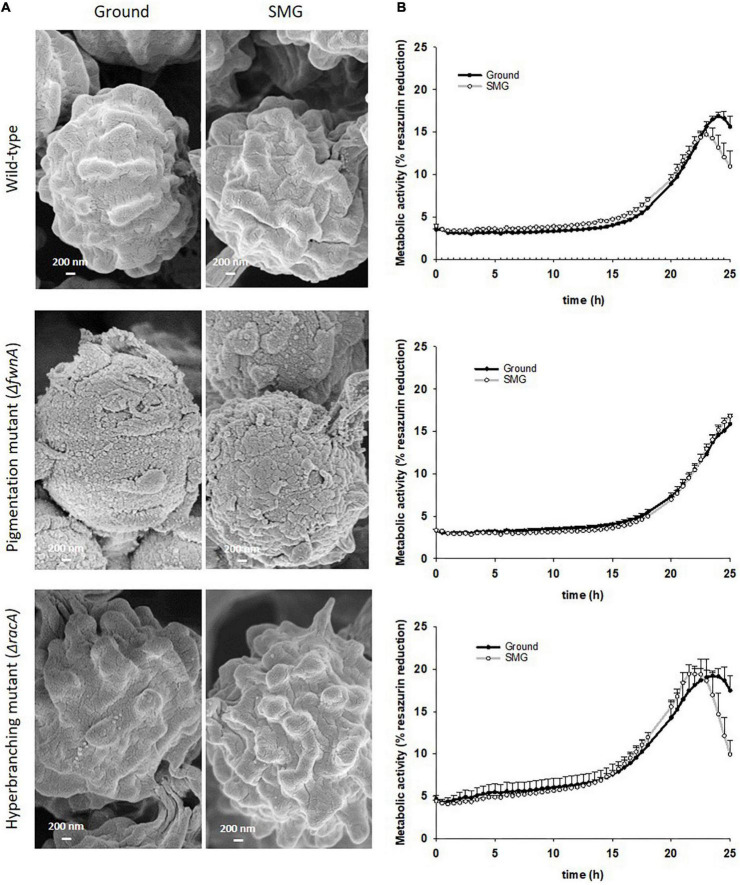
Comparison of spores produced in colonies grown under either Ground or SMG conditions. **(A)** SEM of spores from the central region reveals that the typical surface sculpture of the spores is missing in spores of the pigmentation mutant and that the gravity conditions had no influence on the surface structure. **(B)** Metabolism during spore outgrowth, measured through % reduction of resazurin. Data shown as Mean + SE.

Image analysis of wild-type conidiophores emanating from the central region of the colony showed that they reached, on average, a height of approximately 460 μm high (Figure 2 and Table 2) and displayed normal morphological characteristics for conidiogenesis (Tiedt, 1993): vesicles that develop from stalks formed primary sterigmata (metulae), secondary sterigmata (phialide) and chains of spores which are surrounded with an undulated surface coating consisting of melanin and hydrophobins (Figures 3A,B,G-I and Supplementary Figure 1). Remarkably, results showed that wild-type hyphae from the central region (oldest part of the colony) are densely embedded in extracellular matrix (ECM) (Figures 3C–D), resulting in a approx. 60 μm thick biofilm, which is not yet present around the young hyphae at the colony edge (Figures 3E–F). SEM images of the pigmentation mutant strain (Δ*fwnA*) revealed conidiophores reaching a height of approximately 450 μm (Figures 2, 5). As expected, SEM images of pigmentation mutant Δ*fwnA* spores revealed a less undulated spore surface defective in the characteristic melanin and hydrophobin layer (Figures 6, 7). Similarly, hyphae from the central region of the Δ*fwnA* mutant were shown to be embedded in ECM, resulting in a biofilm thickness of approx. 70 μm.

**FIGURE 8:**
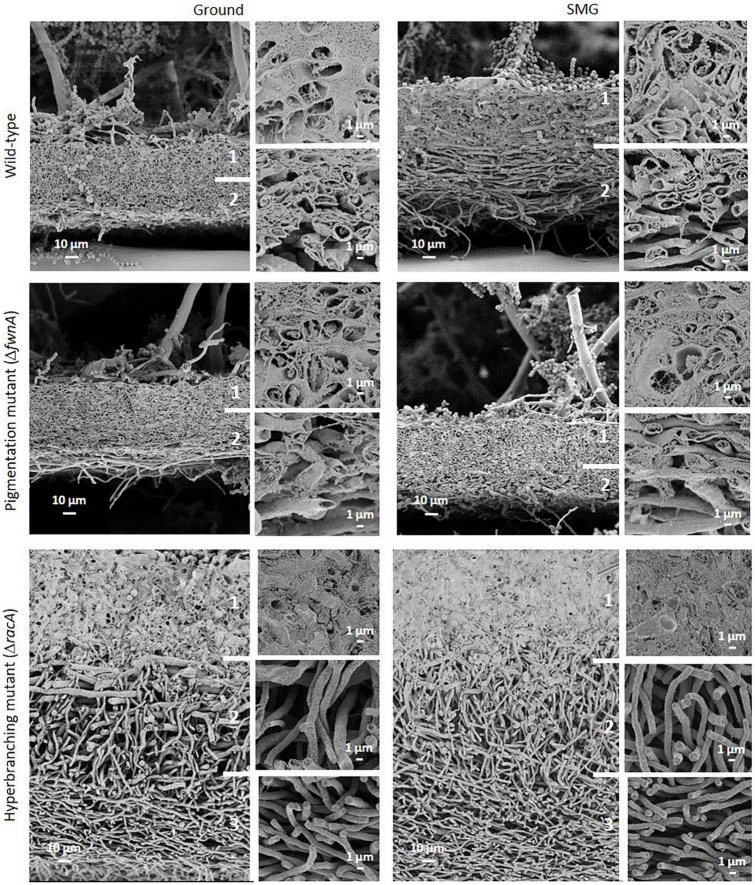
Comparison of A. niger vegetative mycelium (biofilm) from colonies cultivated either under Ground or SMG conditions. A two-layered vegetative mycelium can be seen in the wild-type and pigmentation mutant strainŰan upper layer with hyphae embedded in a dense but porous matrix (ECM), and a lower layer with loose hyphae showing matrix only directly around individual hyphae. The thick mycelium of the hyperbranching mutant strain shows three distinct layers (1–3). (1) Hyphae embedded in dense ECM; (2) vertically oriented hyphae, with no matrix; and (3) horizontally oriented hyphae and no matrix.

Interestingly, SEM images of the hyperbranching mutant strain Δ*racA* revealed that the vegetative mycelium (or biofilm) formed in the central region is approx. 175 μm, which is, remarkably, 3-times thicker than the vegetative mycelium of the wild-type or the Δ*fwnA* mutant strains (Figures 5, 8 and Supplementary Figure 3). The increased thickness of Δ*racA* mycelium would explain the similar dry biomass values compared to the wild-type despite its reduced colony diameter (Figure 2). The morphological structure of the vegetative mycelium in the central region also differed between strains. The vegetative mycelia of wild-type and pigmentation mutant Δ*fwnA* strain both had an extracellular matrix (ECM) that consisted of two distinct layers: an upper layer with hyphae embedded in dense and porous ECM, and a lower layer with loose hyphae and with a thin ECM that coated only individual hyphae (Figure 8). In contrast, the vegetative mycelium of the Δ*racA* mutant showed three distinct layers: an upper layer (63 ± 2.7 μm thick), composed of hyphae embedded in a dense ECM; a middle layer (87 ± 3.5 μm thick), composed of vertically oriented hyphae that are not embedded in an ECM; and a lower layer (45 ± 1.6 μm thick), also with loose but horizontally oriented hyphae, which were not embedded in an ECM (Figure 8). A closer look at the hyperbranching Δ*racA* mutant showed that, independently of the gravitational regime, it formed a folded vegetative mycelium from which aerial hyphae and conidiophores emanated in both directions, i.e., upward and downward (Supplementary Figure 3). Conidiophore stalks of Δ*racA* were also found to be significantly shorter than the wild-type, by 61 % (*p* = 0.002). Besides, conidiophore stalks of Δ*racA* were often able to bud off spores, regardless of the gravitational regime (Supplementary Figure 1), a phenomenon which we only seldomly observed in the wild-type and Δ*fwnA* strains. Furthermore, it became evident that the hyperbranching mutant Δ*racA* formed shorter spore chains, on often malformed vesicles (Figures 5, 6 and Supplementary Figure 2).

### Colony growth of *Aspergillus niger* strains under simulated microgravity

A summary of simulated microgravity (SMG) induced changes revealed in this study can be found in Table 2, for all tested strains. As a first assessment, possible changes in colony growth were determined by measuring colony area, dried biomass as well as the number of spores produced. Results reported that all tested strains registered a slight increase in colony diameter when cultivated under SMG conditions (Figure 2). Wild-type colonies showed a 12 % increase in colony area in SMG (*p* = 0.3), with an average area of 3.7 cm^2^, whereas pigmentation mutant Δ*fwnA* colonies showed a significant 17 % increase in colony area under SMG conditions (*p* = 0.07), with an average area of 4.4 cm^2^. Colonies of the hyperbranching mutant Δ*racA* showed only a slight increase of 4% in colony area (*p* = 0.3) when grown in SMG conditions, registering a small average area of 1.3 cm^2^. Further analysis showed that simulated microgravity affected colony biomass in both mutant strains (Figure 2), revealing an increase of 45 % biomass in pigmentation mutant Δ*fwnA* colonies (*p* = 0.2) under SMG conditions, and a 35 % decrease in colony biomass in hyperbranching mutant colonies (*p* = 0.1) under SMG conditions, when compared to Ground-grown colonies. When analyzing spore production, wild-type colonies produced similarly high numbers of spores in both Ground (6.0 x 10^7^ spores) and SMG conditions (7.0 x 10^7^ spores). However, the number of spores produced under SMG conditions were shown to increase significantly in colonies of both mutant strains, with a 26 % increase in pigmentation mutant Δ*fwnA* colonies (*p* = 0.03), and a 35 % increase in hyperbranching mutant Δ*racA* colonies (*p* = 0.03). These results imply that asexual sporulation might occur faster under simulated microgravity conditions, but that this is, however, strain-dependent.

To study the effect of simulated microgravity in colony ultrastructure, scanning electron microscopy (SEM) of colony cross fractures was performed in small fragments of each colony’s central region (radius 0 - 0.4 cm), which is exposed to simulated microgravity values between 1.2 x 10^–2^ to 1.6 x 10^–2^ g. Results of SEM image analysis demonstrated that SMG conditions induced changes in the thickness of the vegetative mycelium at the central region of the colonies. The thickness of the vegetative mycelium increased significantly by 22% in the wild-type (*p* = 0.001), 56 % in the hyperbranching mutant (*p* = 0.04), but only 6 % in the pigmentation mutant (*p* = 0.1) (Figure 2E and Table 2). The three distinct layers of vegetative mycelium of the Δ*racA* mutant were also seen in SMG conditions, with no differences in thickness when compared to Ground: upper layer (59 ± 1.5 μm), middle layer (90 ± 5.2 μm), and lower layer (45 ± 2.4 μm, Figure 8). No changes were detected in spore morphology for either of the three strains under SMG compared to Ground conditions (Figures 2, 6 and Table 2). However, measurements of conidiophore height revealed that wild-type conidiophores were 11 % shorter (*p* = 0.4) when grown in simulated microgravity (SMG), with an average height of 417 μm. Also, conidiophores of hyperbranching mutant Δ*racA* were 6 % shorter under SMG (*p* = 0.7), with an average height of 165 μm. In turn, conidiophores of pigmentation mutant Δ*fwnA* were 12 % higher in colonies grown under SMG conditions than those on Ground, with an average height of 510 μm (*p* = 0.4) (Figure 2).

### Integrity of *Aspergillus niger* spores under ground and SMG conditions

To assess whether simulated microgravity induced changes in spore integrity, we analyzed spore metabolic activity and spore hydrophobicity as integrity indicators. The results are summarized in - Table 2. Metabolic activity of spores produced under simulated microgravity was not significantly altered in any of the tested strains when compared to the metabolic activity of spores produced on Ground, reaching maximum metabolic activity after 24 - 26 h post-inoculation (Figure 7 and Table 2). Nevertheless, some differences between strains could be observed. For instance, under normal gravitational conditions, spores from the pigmentation mutant Δ*fwnA* were shown to reach the metabolic maximum 2 hours later than wild-type spores (*p* = 0.02). And spores from the hyperbranching mutant Δ*racA* had a higher metabolic activity than wild-type spores, both on Ground (14 % more, *p* = 0.03) and in SMG conditions (33 % more, *p* = 0.03) (Figure 7 and Table 2). Furthermore, spore hydrophobicity was detected by determining the percentage of spores in the hydrocarbon phase (Table 2). Results showed that wild-type spores had similar hydrophobicity values regardless of the gravitational regime they were produced in (49 % on Ground and 51% SMG). The same was reported in spores of the pigmentation mutant Δ*fwnA*, with hydrophobicity values of 31% on Ground, and 34% in simulated microgravity (Figure 2 and Table 2). Yet, pigmentation mutant Δ*fwnA* spores were less hydrophobic than wild-type spores, both under Ground (36 %, *p* = 0.02) and simulated microgravity (32 %, *p* = 0.4). In contrast, results showed that spores of the hyperbranching mutant Δ*racA* were significantly more hydrophobic than wild-type spores when produced in simulated microgravity (77 % increase, *p* = 0.07), a trend also seen under Ground conditions (18 % more hydrophobic than wild-type, *p* = 0.6) (Table 2). Additionally, given the structural role of pigments in the spore cell wall, we investigated the effect of a pigmentation deficiency Δ*fwnA* in spore germination rate and whether this was affected in spores produced under simulated microgravity. Interestingly, wild-type spores produced under simulated microgravity had a 42 % decrease in germination rate (5.6 ± 1 G/NG) compared to spores produced on Ground (10 ± 0.4 G/NG) (*p* = 0.02) (Table 2). In pigmentation mutant Δ*fwnA* spores, the germination rate was not affected by simulated microgravity with the same germination rate of 15 G/NG being reported both in Ground and SMG. Interestingly, results showed that a deletion in Δ*fwnA* led to significantly higher spore germination rates than in fully pigmented wild-type spores, regardless of the gravitational regime. This is, in Ground conditions, spores deficient in FwnA-derived pigmentation registered a 52 % higher germination rate than wild-type spores (*p* = 0.01), and similarly, under SMG conditions, spores deficient in FwnA-derived pigmentation registered 165 % higher germination rate than wild-type spores (*p* = 0.03) (Table 2). Overall, results indicate that wild-type spore germination is affected under simulated microgravity, but that there are no significant changes in metabolic activity or spore hydrophobicity (Table 2).

## Discussion

The filamentous fungus *A. niger* is a common colonizer of indoor-habitats such as the ISS, typically contaminating surfaces in the form of colony biofilms. However, the mechanisms of filamentous fungal biofilm maturation are only now beginning to be understood. Recent studies emphasize colony morphology and biofilm architecture, as valuable indicators for fungal physiology (Kowalski et al., 2021). Thus, in this study, we investigated *A. niger* colony growth and biofilm formation on agar, as an analog to fungal surface contamination, under normal gravitational conditions (Ground) and simulated microgravity (SMG). Three *A. niger* strains were included: a fully-pigmented wild-type strain, a pigmentation mutant strain (Δ*fwnA*), and a hyperbranching mutant strain (Δ*racA*) of biotechnological interest.

Our study reveals never-before seen scanning electron microscopy images of colony cross fractures that provide insights into fungal wild-type colony development under normal gravitational conditions. To our knowledge, only the review of Krijgsheld et al. (2013) reported fungal macromorphology via SEM imaging of an *A. niger* 7-day colony grown on agar, showing the transition from single hyphal layer to a hyphae multilayer. However, because the colony was grown sandwiched between two polycarbonate membranes, conidiation and sporulation were prevented, and resulted in compromised colony growth. In contrast, our study presents SEM images of a fungal colony in its entirety, not only revealing the complex cellular network and intricate ultrastructure of the vegetative mycelium (biofilm) through which *A. niger* colonizes its surrounding environment; but also depicting important reproductive structures of the fungal aerial mycelium, i.e., the conidiophores and asexual spores (i.e., conidia), and most importantly, exposing the interface between vegetative and aerial mycelium.

SEM analysis of *A. niger* wild-type colonies show that the youngest part of the colony (at the very edge) is composed of a single layer of hyphae with an intact cytoplasm. In turn, hyphae at the oldest and most matured part of the colony (at the innermost center) are arranged in a complex multilayer and have a rather empty and intact cytoplasm, with no structural signatures. We propose that young hyphae of *A. niger* mainly work as nutrient scavengers, having a highly active and compact cytoplasm, whereas old hyphae are important in setting the biofilm’s three-dimensional structure, where only the cell wall is needed and thus the cytoplasm appears empty. Mature fungal colonies have been reported to be composed of both metabolic active, metabolic inactive as well as dead hyphae, the latter of which develop through autophagy (Boswell and Hopkins, 2008; Krijgsheld et al., 2013, Emri et al., 2018; Kaur and Punekar, 2019; Khalid et al., 2019; Lingo et al., 2021). Our study supports these observations and further highlights that young hyphae at the edge of the colony are not significantly covered by an extracellular matrix. This is also in agreement with the loose mycelial network of *A. niger* reported by Krijgsheld et al., 2013 that is different from the mycelial network in the innermost center of the colony, which is at least partially, but often integrally, embedded in a dense ECM. In the mature colony center, the dense ECM is strongly present in the upper layer of the vegetative mycelium. In bacterial biofilms, the ECM acts as a shield from environmental stressors, including cleaning agents and antimicrobials (Dragoš and Kovács, 2017). However, young active hyphae at the colony edge appear rather unprotected. Therefore, we propose that, in filamentous fungi, the main function of the dense ECM is to act as structural support for the formation of the aerial mycelium. By depositing in the center of the colony, the ECM mechanically stabilizes the mycelium of the dying hyphae and provides support for the developing conidiophores and spores. The mechanical stabilization of ECM surrounded hyphae is likely also aided by water storage in the “empty” inactive hyphae. Further quantitative analysis of the SEM images showed that *A. niger* wild-type conidiophores can reach an average height of ∼ 460 μm, which is four times higher than initially reported, for example in *A. nidulans* (100 μm) (Adams et al., 1998), and suggests an efficient air-dispersal ability of *A. niger*’s spores that contributes to the colonization of different habitats. Given the results of our study, *A. niger* wild-type colony growth under static, aerial conditions, regardless of the gravitational regime, can be summarized as follows: 1) spore germination and outgrowth followed by hyphal expansion and increase in colony area; 2) hyphae multilayering and increase in vegetative mycelium thickness; 3) increased secretion of ECM and maturation into a biofilm; 4) cell death (supposedly via autophagy) and transition from active hyphae (nutrient scavenging) to inactive hyphae (structural); 5) formation of aerial hyphae, conidiophores and sporulation.

Our study shows that simulated microgravity affects *A. niger* colony area, spore production and biofilm thickness in a strain-dependent manner. Most importantly, we show that the growth of *A. niger* is not inhibited by simulated microgravity by fast-clinorotation of 60 rpm in a 2-D clinostat. However, our findings suggest *A. niger*’s contamination potential of spaceflight environments might increase, given the simulated microgravity increase in spore production and biofilm thickness. In particular, the formation of significantly thicker wild-type biofilms under SMG indicates increased potential for surface colonization and consequently also material biodegradation. This can be of concern for spacecraft materials, air- and water systems, and should be addressed in future studies. Exactly how fungi adapt to different gravitational conditions is still not well understood (Häder, 2018). To our knowledge, gravity sensing mechanisms in fungi have so far been identified in Phycomyces, a mucoralean fungus that uses the sedimentation of vacuolar protein crystals and the buoyancy of lipid globules in the hyphal apices to direct the gravitropism of its sporangiophores (Moore, 1996, Schimek et al., 1999, Grolig et al., 2006). Previous studies also reported actin polymerization as central to the growth of *Saccharomyces cereviseae* in a bioreactor under microgravity conditions (Walther et al., 1996).

In this study we provide valuable insights on the mechanisms of fungal adaptation to spaceflight microgravity. For instance, we suggest the role of the Rho GTPase RacA in *A. niger*’s adaptation to simulated microgravity, as its deletion leads to increased biofilm thickness, increased spore production and decreased total biomass of colonies grown in SMG conditions. Both wild-type and Δ*racA* mutant form thicker biofilms under simulated microgravity, which could, in part, be due to potential changes in nutrient availability facilitated by the simulated microgravity environment, that would affect general colony growth (such as colony area and dried biomass) (Rosenzweig et al., 2010; Bizzarri et al., 2015). However, deletion in Δ*racA* formed three times thicker biofilms than the wild-type, probably due to its phenotype of hyperbranching and hypersecretion of extracellular products (Fiedler et al., 2018). RacA controls actin polymerization in *A. niger* and is thus responsible for maintaining hyphal tip polarity and hyphal branch initiation – both are essential for the establishment of the complex hyphal network characteristic for a fungal colony biofilm (Fiedler et al., 2018). Actin is furthermore known to control several cellular processes, such as intracellular movement of organelles, vesicular trafficking and cytokinesis (Walker and Garrill, 2006; Bergs et al., 2016). Actin has also been proposed as a graviperception facilitator (Velle and Fritz-Laylin, 2019; Fajardo-Cavazos and Nicholson, 2021) since it is involved in re-orientation of direction of movement or growth. We suggest that RacA-mediated actin-controlled polar growth can be involved in the response to simulated microgravity in *A. niger* by stimulating hyphal growth and ECM production, which in turn result in an increased thickness of the vegetative mycelium. Because RhoGTPases were found to be important in mammalian cell adaptation to microgravity (Louis et al., 2015), thus, it is likely that also RacA is a key-element for filamentous fungi adaptation to a low gravitational regime.

We furthermore propose that FwnA-mediated melanin production is involved in the response to simulated microgravity, as Δ*fwnA* colonies showed increased growth and spore production under SMG conditions. Secondary metabolites, such as pigments, are known to be involved a wide-range of cellular processes, from protection from environmental stress (e.g. radiation, ROS, and possibly microgravity) to pathogenicity (Cordero and Casadevall, 2017; Lin and Xu, 2020). *A. niger* pigments, such as melanins, are an important part of the spore surface coating, but are also thought to be present in the hyphae and extracellular matrix (Cortesão et al., 2020a; Lin and Xu, 2020). Interestingly, Sun et al. (2021) suggested the impact of melanin biosynthesis genes *Abr1* and *Ayg1* in *A. niger* biofilm production, where mycelial melanin production is thought to be mediated by the MAPK signaling pathway via activation of the *RlmA* transcription factor. Deletion of the polyketide synthase FwnA in *A. niger* leads to the production of fawn-colored spores (Jørgensen et al., 2011), in contrast to white colored spores produced upon deletion of the homolog Alb1 (or PKSP) in *A. fumigatus* (Pihet et al., 2009, Bayry et al., 2014). Whether *A. niger* spore melanin is formed through the DHN pathway is still a matter of debate. Nevertheless, in this study, SEM images reveal, as expected, that *A. niger ΔfwnA* spores have less undulated spore surface coating, but are not completely devoid of them, which is similar to data obtained from the Δ*ayg1* or Δ*arp2* mutants in *A. fumigatus* (Pihet et al., 2009; Bayry et al., 2014). Furthermore, we suggest that FwnA-mediated pigmentation plays a role in *A. niger* biofilm formation, as lack of Δ*fwnA* leads to significantly thicker biofilms, and lower biomass than the wild-type. Lack of Δ*fwnA* also leads to a significantly higher spore production than the wild-type, at least in the first 3 days of colony growth, under both Ground and SMG conditions. Interestingly, a previous study suggested that blocking the melanin biosynthetic pathway may result in an accumulation of the precursor substrate acetyl-CoA which may cause the increase in sporulation in the fungus *Pestalotiopsis microspora* (Yu et al., 2015). Such a putative excess of acetyl-CoA might also help *A. niger* to faster colonize the environment under microgravity conditions, a hypothesis worth studying further.

Taken together, our study shows that *A. niger* growth is not inhibited and spore integrity is not hindered by simulated microgravity, but rather indicates a potential increase in surface-colonization, due to the formation of thicker biofilms and increased spore production. By revealing the complex morphology and ultrastructure of the *A. niger* biofilm, and reporting its surface-associated growth under simulated microgravity conditions, our study contributes to the fields of fungal research, healthcare and aerospace. In particular, given the natural presence of *A. niger* spores in indoor-habitats (from hospitals to airplane cabins and space stations), and considering the status of the fungus as opportunistic human pathogenic. We thus emphasize the need to further study fungal growth and surface contaminations to help develop strategies and technologies that control and mitigate fungal biofilms, on Earth and in space. Finally, we note the importance of investigating filamentous fungi in the context of long-term space missions, not only to reduce the risk of negatively impacting human health and spacecraft material safety, but also to positively utilize fungal-based biotechnology to acquire needed resources in situ (Zea et al., 2020, Cortesão et al., 2020b,Santomartino et al., 2022).

## Data availability statement

The raw data supporting the conclusions of this article will be made available by the authors, without undue reservation.

## Author contributions

MC designed and performed the experiments and analyzed the data. GH and ML assisted with SEM experimental design and supervised sample preparation, imaging, and analysis. MC, TS, VM, and RM designed the study and jointly interpreted the data. MC and VM co-wrote the manuscript which was approved by all co-authors. All authors contributed to the article and approved the submitted version.

## References

[B1] Adams T. H. Wieser J. K. Yu J. H. (1998). Asexual sporulation in *Aspergillus nidulans*. *Microbiol. Mol. Biol. Rev.* 62 35–54. 10.1128/MMBR.62.1.35-54.1998 9529886 PMC98905

[B2] Anken R. (2013). Simulation of microgravity for studies in gravitational biology: Principles, devices and applications. *Curr. Biotechnol.* 2:3. 10.2174/22115501113029990012

[B3] Bayry J. Beaussart A. Dufrêne Y. F. Sharma M. Bansal K. Kniemeyer O. (2014). Surface structure characterization of *Aspergillus fumigatus* conidia mutated in the melanin synthesis pathway and their human cellular immune response. *Infect. Immun.* 82 3141–3153. 10.1128/IAI.01726-14 24818666 PMC4136205

[B4] Beauvais A. Fontaine T. Aimanianda V. Latgé J. P. (2014). *Aspergillus* cell wall and biofilm. *Mycopathologia* 178 371–377. 10.1007/s11046-014-9766-0 24947169

[B5] Beauvais A. Latgé J.-P. (2015). *Aspergillus* biofilm in vitro and in vivo. *Microbiol. Spectr.* 3 MB–0017. 10.1128/microbiolspec.MB-0017-2015 26350307

[B6] Beauvais A. Schmidt C. Guadagnini S. Roux P. Perret E. Henry C. (2007). An extracellular matrix glues together the aerial-grown hyphae of *Aspergillus fumigatus*. *Cell. Microbiol.* 9 1588–1600. 10.1111/j.1462-5822.2007.00895.x 17371405

[B7] Bergs A. Ishitsuka Y. Evangelinos M. Nienhaus G. U. Takeshita N. (2016). Dynamics of actin cables in polarized growth of the filamentous fungus *Aspergillus nidulans*. *Front. Microbiol.* 7:682. 10.3389/fmicb.2016.00682 27242709 PMC4860496

[B8] Bizzarri M. Monici M. Loon J. J. W. A. V. (2015). How microgravity affects the biology of living systems. *Biomed. Res. Int.* 2015:863075. 10.1155/2015/863075 25667927 PMC4312564

[B9] Blachowicz A. Chiang A. J. Elsaesser A. Kalkum M. Ehrenfreund P. Stajich J. E. (2019). Proteomic and metabolomic characteristics of extremophilic fungi under simulated Mars conditions. *Front. Microbiol.* 10:1013. 10.3389/fmicb.2019.01013 PMC6529585 31156574

[B10] Bos C. J. Debets A. J. M. Swart K. Huybers A. Kobus G. Slakhorst S. M. (1988). Genetic analysis and the construction of master strains for assignment of genes to six linkage groups in *Aspergillus niger*. *Curr. Genet.* 14 437–443. 10.1007/BF00521266 3224384

[B11] Boswell G. P. Hopkins S. (2008). Linking hyphal growth to colony dynamics: Spatially explicit models of mycelia. *Fungal Ecol.* 1 143–154. 10.1016/j.funeco.2008.10.003

[B12] Cairns T. C. Nai C. Meyer V. (2018). How a fungus shapes biotechnology: 100 years of *Aspergillus niger* research. *Fungal Biol. Biotechnol.* 5:13. 10.1186/s40694-018-0054-5 29850025 PMC5966904

[B13] Chung K. Y. Brown J. C. S. (2020). Biology and function of exo-polysaccharides from human fungal pathogens. *Curr. Clin. Microbiol. Rep.* 7 1–11. 10.1007/s40588-020-00137-5 33042730 PMC7543870

[B14] Cordero R. J. Casadevall A. (2017). Functions of fungal melanin beyond virulence. *Fungal Biol. Rev.* 31 99–112. 10.1016/j.fbr.2016.12.003 31649746 PMC6812541

[B15] Cortesão M. de Haas A. Unterbusch R. Fujimori A. Schütze T. Meyer V. (2020a). *Aspergillus niger* spores are highly resistant to space radiation. *Front. Microbiol.* 11:560. 10.3389/fmicb.2020.00560 32318041 PMC7146846

[B16] Cortesão M. Schütze T. Marx R. Moeller R. Meyer V. (2020b). “Fungal biotechnology in space: Why and how?,” in *Grand challenges in fungal biotechnology. Grand challenges in biology and biotechnology*, ed. Nevalainen H. (Cham: Springer), 10.1007/978-3-030-29541-7_18

[B17] Di Pippo F. Di Gregorio L. Congestri R. Tandoi V. Rossetti S. (2018). Biofilm growth and control in cooling water industrial systems. *FEMS Microbiol. Ecol.* 94:fiy044. 10.1093/femsec/fiy044 29596620

[B18] Dragoš A. Kovács A. T. (2017). The peculiar functions of the bacterial extracellular matrix. *Trends Microbiol.* 25 257–266. 10.1016/j.tim.2016.12.010 28089324

[B19] Eiermann P. Kopp S. Hauslage J. Hemmersbach R. Gerzer R. Ivanova K. (2013). Adaptation of a 2-D clinostat for simulated microgravity experiments with adherent cells. *Microgravity Sci. Technol.* 25 153–159. 10.1007/s12217-013-9341-1

[B20] Emri T. Vékony V. Gila B. Nagy F. Forgács K. Pócsi I. (2018). Autolytic hydrolases affect sexual and asexual development of *Aspergillus nidulans*. *Folia Microbiol. (Praha)* 63 619–626. 10.1007/s12223-018-0601-8 29603054

[B21] Faille C. Ronse A. Dewailly E. Slomianny C. Maes E. Krzewinski F. (2014). Presence and function of a thick mucous layer rich in polysaccharides around *Bacillus subtilis* spores. *Biofouling* 30 845–858. 10.1080/08927014.2014.939073 25115519

[B22] Fajardo-Cavazos P. Nicholson W. (2021). Mechanotransduction in prokaryotes: A possible mechanism of spaceflight adaptation. *Life (Basel)* 11:33. 10.3390/life11010033 33430182 PMC7825584

[B23] Fengler S. Spirer I. Neef M. Ecke M. Hauslage J. Hampp R. (2016). Changes in gene expression of *Arabidopsis thaliana* cell cultures upon exposure to real and simulated partial-g forces. *Microgravity Sci. Technol.* 28 319–329. 10.1007/s12217-015-9452-y

[B24] Fiedler M. R. M. Barthel L. Kubisch C. Nai C. Meyer V. (2018). Construction of an improved *Aspergillus niger* platform for enhanced glucoamylase secretion. *Microb. Cell. Fact.* 17:95. 10.1186/s12934-018-0941-8 29908567 PMC6004097

[B25] Fuchs F. M. Holland G. Moeller R. Laue M. (2018). Directed freeze-fracturing of *Bacillus subtilis* biofilms for conventional scanning electron microscopy. *J. Microbiol. Methods* 152 165–172. 10.1016/j.mimet.2018.08.005 30125587

[B26] Garschagen L. S. Mancinelli R. L. Moeller R. (2019). Introducing Vibrio natriegens as a microbial model organism for microgravity research. *Astrobiology* 19 1211–1220. 10.1089/ast.2018.2010 31486680

[B27] Gilbert R. Torres M. Clemens R. Hateley S. Hosamani R. Wade W. (2020). Spaceflight and simulated microgravity conditions increase virulence of *Serratia marcescens* in the *Drosophila melanogaster* infection model. *npj Microgravity* 6:4. 10.1038/s41526-019-0091-2 32047838 PMC7000411

[B28] Gomoiu I. Chatzitheodoridis E. Vadrucci S. Cojoc R. (2016). Fungal spores viability on the international space station. *Orig. Life Evol. Biosph.* 46 403–418. 10.1007/s11084-016-9502-5 27106019

[B29] Gomoiu I. Chatzitheodoridis E. Vadrucci S. Walther I. (2013). The effect of spaceflight on growth of *Ulocladium chartarum* colonies on the international space station. *PLoS One* 8:e62130. 10.1371/journal.pone.0062130 23637980 PMC3634740

[B30] Grolig F. Döring M. Galland P. (2006). Gravisusception by buoyancy: A mechanism ubiquitous among fungi? *Protoplasma* 229 117–123. 10.1007/s00709-006-0218-7 17180492

[B31] Gutarowska B. (2010). Metabolic activity of moulds as a factor of building materials biodegradation. *Polish J. Microbiol.* 59 119–124. 20734757

[B32] Häder D. P. (2018). “Gravitropism in fungi, mosses and ferns,” in *Gravitational biology I: Gravity sensing and graviorientation in microorganisms and plants*, eds Braun M. Böhmer M. Häder D.-P. Hemmersbach R. Palme K. (Cham: Springer International Publishing), 67–74. 10.1007/978-3-319-93894-3

[B33] Hammond T. G. Stodieck L. Birdsall H. H. Becker J. L. Koenig P. Hammond J. S. (2013). Effects of microgravity on the virulence of *Listeria monocytogenes*, *Enterococcus faecalis*, *Candida albicans*, and methicillin-resistant *Staphylococcus aureus*. *Astrobiology* 13 1081–1090. 10.1089/ast.2013.0986 24283929

[B34] Hasenstein K. H. van Loon J. J. W. A. (2015). “Clinostats and other rotating systems-design, function, and limitations,” in *Generation and applications of extra-terrestrial environments on earth*, eds Beysens D. A. van Loon J. J. W. A. (Delft: River Publishers), 147–156.

[B35] Herranz R. Anken R. Boonstra J. Braun M. Christianen P. C. M. de Geest M. (2013). Ground-based facilities for simulation of microgravity: Organism-specific recommendations for their use, and recommended terminology. *Astrobiology* 13 1–17. 10.1089/ast.2012.0876 23252378 PMC3549630

[B36] Horneck G. Klaus D. M. Mancinelli R. L. (2010). Space microbiology. *Annu. Rev. Microbiol.* 74 121–137. 10.1128/MMBR.00016-09 20197502 PMC2832349

[B37] Jiang C. Guo D. Li Z. Lei S. Shi J. Shao D. (2019). Clinostat rotation affects metabolite transportation and increases organic acid production by *Aspergillus carbonarius*, as revealed by differential metabolomic analysis. *Appl. Environ. Microbiol.* 85 1–45. 10.1128/AEM.01023-19 31300399 PMC6715838

[B38] Jørgensen T. R. Park J. Arentshorst M. van Welzen A. M. Lamers G. Vankuyk P. A. (2011). The molecular and genetic basis of conidial pigmentation in *Aspergillus niger*. *Fungal Genet. Biol.* 48 544–553. 10.1016/j.fgb.2011.01.005 21277986

[B39] Kaur B. Punekar N. S. (2019). Autophagy is important to the acidogenic metabolism of *Aspergillus niger*. *PLoS One* 14:e0223895. 10.1371/journal.pone.0223895 31603923 PMC6788731

[B40] Khalid A. R. Lv X. Naeem M. Mehmood K. Shaheen H. Dong P. (2019). Autophagy related gene (ATG3) is a key regulator for cell growth, development, and virulence of *Fusarium oxysporum*. *Genes (Basel)* 10:658. 10.3390/genes10090658 31466418 PMC6769740

[B41] Kikuma T. Arioka M. Kitamoto K. (2007). Autophagy during conidiation and conidial germination in filamentous fungi. *Autophagy* 3 128–129. 10.4161/auto.3560 17183223

[B42] Klaus D. M. (2001). Clinostats and bioreactors. *Gravit. Space Biol. Bull.* 14 55–64. 11865869

[B43] Klintworth R. Reher H. J. Viktorov A. N. Bohle D. (1999). Biological induced corrosion of materials II: New test methods and experiences from MIR station. *Acta Astronaut.* 44 569–578. 10.1016/s0094-5765(99)00069-7 11542520

[B44] Kowalski C. H. Morelli K. A. Stajich J. E. Nadell C. D. Cramer R. A. (2021). A heterogeneously expressed gene family modulates the biofilm architecture and hypoxic growth of *Aspergillus fumigatus.* *mBio* 12:e03579Ű20. 10.1128/mBio.03579-20 33593969 PMC8545126

[B45] Krause L. Braun M. Hauslage J. Hemmersbach R. (2018). Analysis of statoliths displacement in chara rhizoids for validating the microgravity-simulation quality of clinorotation modes. *Microgravity Sci. Technol.* 30 229–236. 10.1007/s12217-017-9580-7

[B46] Krijgsheld P. Bleichrodt R. van Veluw G. J. Wang F. Müller W. H. Dijksterhuis J. (2013). Development in *Aspergillus*. *Stud. Mycol.* 74 1–29. 10.3114/sim0006 23450714 PMC3563288

[B47] Kwon M. J. Arentshorst M. Roos E. D. van den Hondel C. A. M. J. J. Meyer V. Ram A. F. J. (2011). Functional characterization of Rho GTPases in *Aspergillus niger* uncovers conserved and diverged roles of Rho proteins within filamentous fungi. *Mol. Microbiol.* 79 1151–1167. 10.1111/j.1365-2958.2010.07524.x 21205013

[B48] Lagree K. Mitchell A. P. (2017). Fungal biofilms: Inside out. *Fungal Kingdom* 5 873–886. 10.1128/microbiolspec.FUNK-0024-2016 28387175 PMC5972825

[B49] Lin L. Xu J. (2020). Fungal pigments and their roles associated with human health. *J. Fungi (Basel Switzerland)* 6:280. 10.3390/jof6040280 33198121 PMC7711509

[B50] Lingo D. E. Shukla N. Osmani A. H. Osmani S. A. (2021). *Aspergillus nidulans* biofilm formation modifies cellular architecture and enables light-activated autophagy. *Mol. Biol. Cell* 32 1181–1192. 10.1091/mbc.E20-11-0734 33826367 PMC8351559

[B51] Louis F. Deroanne C. Nusgens B. Vico L. Guignandon A. (2015). RhoGTPases as key players in mammalian cell adaptation to microgravity. *Biomed. Res. Int.* 2015:747693. 10.1155/2015/747693 25649831 PMC4310447

[B52] Makimura K. Hanazawa R. Takatori K. Tamura Y. Fujisaki R. Nishiyama Y. (2001). Fungal flora on board the Mir-space station, identification by morphological features and ribosomal DNA sequences. *Microbiol. Immunol.* 45 357–363. 10.1111/j.1348-0421.2001.tb02631.x 11471823

[B53] Mela A. P. Rico-Ramírez A. M. Glass N. L. (2020). Syncytia in fungi. *Cells* 9:2255. 10.3390/cells9102255 33050028 PMC7600787

[B54] Meyer V. Basenko E. Y. Wösten H. A. B. (2020). Growing a circular economy with fungal biotechnology: A white paper. *Fungal Biol. Biotechnol.* 7:5. 10.1186/s40694-020-00095-z 32280481 PMC7140391

[B55] Moore D. (1996). Graviresponses in fungi. *Adv. Sp. Res.* 17 73–82. 10.1016/0273-1177(95)00614-k 11538639

[B56] National Research Council (Us) Committee on Air Quality in Passenger Cabins of Commercial Aircraft (2002). *The airliner cabin environment and the health of passengers and crew.* Washington, DC: National Academies Press, 10.17226/10238 25032286

[B57] Nitsche B. M. Burggraaf-van Welzen A. M. Lamers G. Meyer V. Ram A. F. J. (2013). Autophagy promotes survival in aging submerged cultures of the filamentous fungus *Aspergillus niger*. *Appl. Microbiol. Biotechnol.* 97 8205–8218. 10.1007/s00253-013-4971-1 23700238

[B58] Novikova N. Boever P. D. Poddubko S. Deshevaya E. Polikarpov N. Rakova N. (2006). Survey of environmental biocontamination on board the International Space Station. *Res. Microbiol.* 157 5–12. 10.1016/j.resmic.2005.07.010 16364606

[B59] Novikova N. D. (2004). Review of the knowledge of microbial contamination of the Russian manned spacecraft. *Microb. Ecol.* 47 127–132. 10.1007/s00248-003-1055-2 14994178

[B60] Ott E. Fuchs F. M. Moeller R. Hemmersbach R. Kawaguchi Y. Yamagishi A. (2019). Molecular response of *Deinococcus radiodurans* to simulated microgravity explored by proteometabolomic approach. *Sci. Rep.* 9:18462. 10.1038/s41598-019-54742-6 31804539 PMC6895123

[B61] Pihet M. Vandeputte P. Tronchin G. Renier G. Saulnier P. Georgeault S. (2009). Melanin is an essential component for the integrity of the cell wall of *Aspergillus fumigatus* conidia. *BMC Microbiol.* 9:177. 10.1186/1471-2180-9-177 19703288 PMC2740851

[B62] Priegnitz B. E. Wargenau A. Brandt U. Rohde M. Dietrich S. Kwade A. (2012). The role of initial spore adhesion in pellet and biofilm formation in *Aspergillus niger*. *Fungal Genet. Biol.* 49 30–38. 10.1016/j.fgb.2011.12.002 22178638

[B63] Romsdahl J. Blachowicz A. Chiang A. J. Singh N. Stajich J. E. Kalkum M. (2018). Characterization of *Aspergillus niger* isolated from the International space station. *mSystems* 3 e112–e118. 10.1128/mSystems.00112-18 30246146 PMC6143729

[B64] Rosenzweig J. A. Abogunde O. Thomas K. Lawal A. Nguyen Y. Sodipe A. (2010). Spaceflight and modeled microgravity effects on microbial growth and virulence. *Appl. Microbiol. Biotechnol.* 85 885–891. 10.1007/s00253-009-2237-8 19847423 PMC2804794

[B65] Santomartino R. Zea L. Cockell C. S. (2022). The smallest space miners: Principles of space biomining. *Extremophiles* 26:7. 10.1007/s00792-021-01253-w 34993644 PMC8739323

[B66] Sathishkumar Y. Velmurugan N. Lee H. M. Rajagopal K. Im C. K. Lee Y. S. (2014). Effect of low shear modeled microgravity on phenotypic and central chitin metabolism in the filamentous fungi *Aspergillus niger* and *Penicillium chrysogenum*. *Antonie van Leeuwenhoek* 106 197–209. 10.1007/s10482-014-0181-9 24803238

[B67] Schimek C. Eibel P. Grolig F. Horie T. Ootaki T. Galland P. (1999). Gravitropism in phycomyces: A role for sedimenting protein crystals and floating lipid globules. *Planta* 210 132–142. 10.1007/s004250050662 10592041

[B68] Sheppard D. C. Howell P. L. (2016). Biofilm exopolysaccharides of pathogenic fungi: Lessons from bacteria. *J. Biol. Chem.* 291 12529–12537. 10.1074/jbc.R116.720995 27129222 PMC4933471

[B69] Shuster B. Khemmani M. Abe K. Huang X. Nakaya Y. Maryn N. (2019). Contributions of crust proteins to spore surface properties in *Bacillus subtilis*. *Mol. Microbiol.* 111 825–843. 10.1111/mmi.14194 30582883 PMC6417949

[B70] Sugui J. A. Kwon-Chung K. J. Juvvadi P. R. Latgé J.-P. Steinbach W. J. (2014). *Aspergillus fumigatus* and related species. *Cold Spring Harb. Perspect. Med.* 5:a019786. 10.1101/cshperspect.a019786 25377144 PMC4315914

[B71] Sun W. Yu Y. Chen J. Yu B. Chen T. Ying H. (2021). Light signaling regulates *Aspergillus niger* biofilm formation by affecting melanin and extracellular polysaccharide biosynthesis. *mBio* 12:e03434Ű20. 10.1128/mBio.03434-20 33593965 PMC8545115

[B72] Taylor P. W. (2015). Impact of space flight on bacterial virulence and antibiotic susceptibility. *Infect. Drug Resist.* 8 249–262. 10.2147/IDR.S67275 26251622 PMC4524529

[B73] Tesei D. Chiang A. J. Kalkum M. Stajich J. E. Mohan G. B. M. Sterflinger K. (2021). Effects of simulated microgravity on the proteome and secretome of the polyextremotolerant black fungus *Knufia* chersonesos. *Front. Genet.* 12:638708. 10.3389/fgene.2021.638708 33815472 PMC8012687

[B74] Tiedt L. R. (1993). An electron microscope study of conidiogenesis and wall formation of conidia of *Aspergillus niger*. *Mycol. Res.* 97 1459–1462.

[B75] Van Mulders S. E. Stassen C. Daenen L. Devreese B. Siewers V. van Eijsden R. G. E. (2011). The influence of microgravity on invasive growth in *Saccharomyces cerevisiae*. *Astrobiology* 11 45–55. 10.1089/ast.2010.0518 21345087

[B76] Velle K. B. Fritz-Laylin L. K. (2019). Diversity and evolution of actin-dependent phenotypes. *Curr. Opin. Genet. Dev.* 58–59 40–48. 10.1016/j.gde.2019.07.016 31466039

[B77] Vesper S. J. Wong W. Kuo C. M. Pierson D. L. (2008). Mold species in dust from the International Space Station identified and quantified by mold-specific quantitative PCR. *Res. Microbiol.* 159 432–435. 10.1016/j.resmic.2008.06.001 18602989

[B78] Walker S. K. Garrill A. (2006). Actin microfilaments in fungi. *Mycologist* 20 26–31.

[B79] Walther I. Bechler B. Müller O. Hunzinger E. Cogoli A. (1996). Cultivation of *Saccharomyces cerevisiae* in a bioreactor in microgravity. *J. Biotechnol.* 47 113–127. 10.1016/0168-1656(96)01375-2 8987565

[B80] Yamaguchi N. Roberts M. Castro S. Oubre C. Makimura K. Leys N. (2014). Microbial monitoring of crewed habitats in space-current status and future perspectives. *Microbes Environ.* 29 250–260. 10.1264/jsme2.ME14031 25130885 PMC4159036

[B81] Yamazaki T. Yoshimoto M. Nishiyama Y. Okubo Y. Makimura K. (2012). Phenotypic characterization of *Aspergillus niger* and *Candida albicans* grown under simulated microgravity using a three-dimensional clinostat. *Microbiol. Immunol.* 56 441–446. 10.1111/j.1348-0421.2012.00471.x 22537211

[B82] Yu X. Huo L. Liu H. Chen L. Wang Y. Zhu X. (2015). Melanin is required for the formation of the multi-cellular conidia in the endophytic fungus *Pestalotiopsis microspora*. *Microbiol. Res.* 179 1–11. 10.1016/j.micres.2015.06.004 26411889

[B83] Zea L. McLean R. J. C. Rook T. A. Angle G. Carter D. L. Delegarde A. (2020). Potential biofilm control strategies for extended spaceflight missions. *Biofilm* 2:100026. 10.1016/j.bioflm.2020.100026 33447811 PMC7798464

