# Supplementary material for: Colony growth and biofilm formation of Aspergillus niger under simulated microgravity
Source: Front Microbiol. 2022 Sep 23;13:975763. doi: 10.3389/fmicb.2022.975763 (PMC9539656; doi:10.3389/fmicb.2022.975763)
Supplement: Supplementary file 1 [file Data_Sheet_1.DOCX]

Supplementary Material

## Supplementary Figures

Pigmentation mutant (∆*fwnA*)


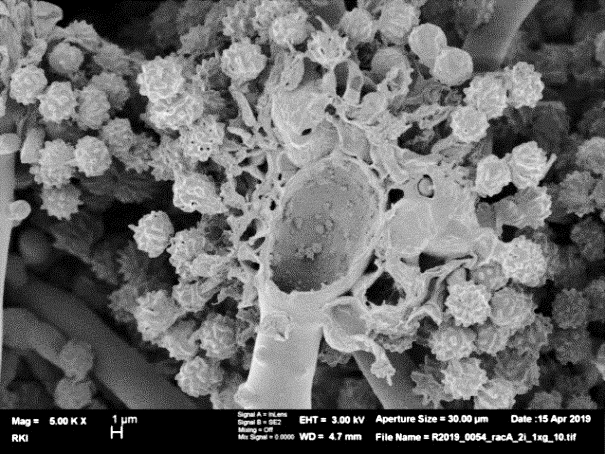

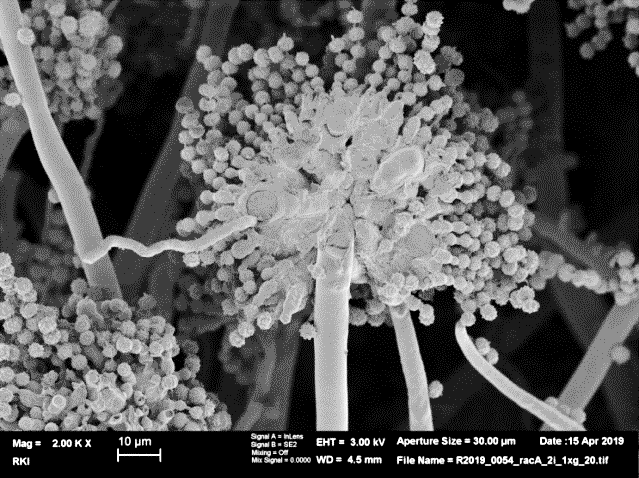

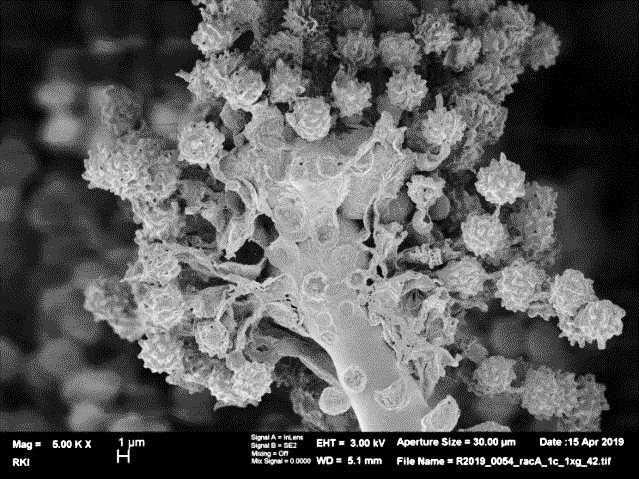

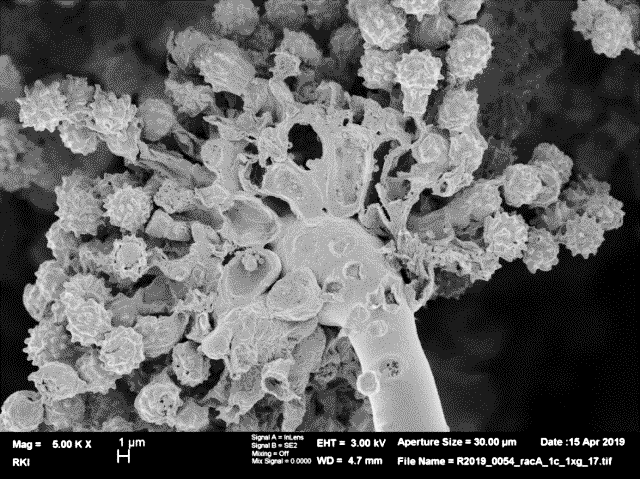


**1 µm**


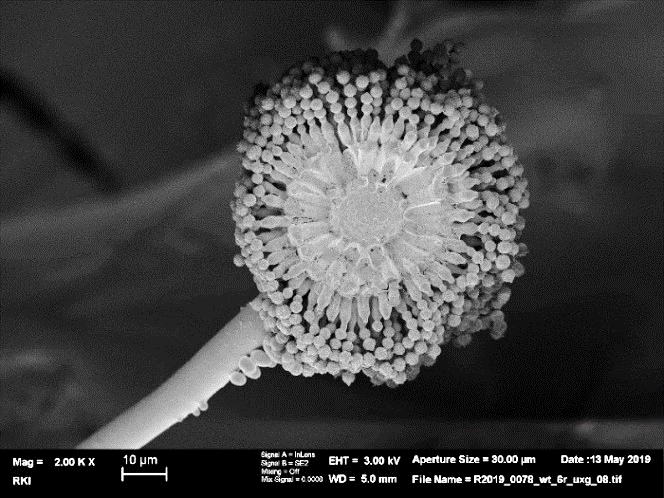

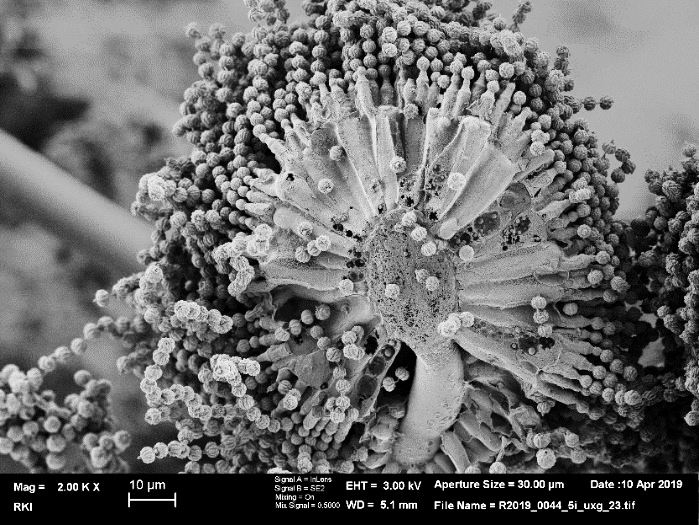


Wild-type


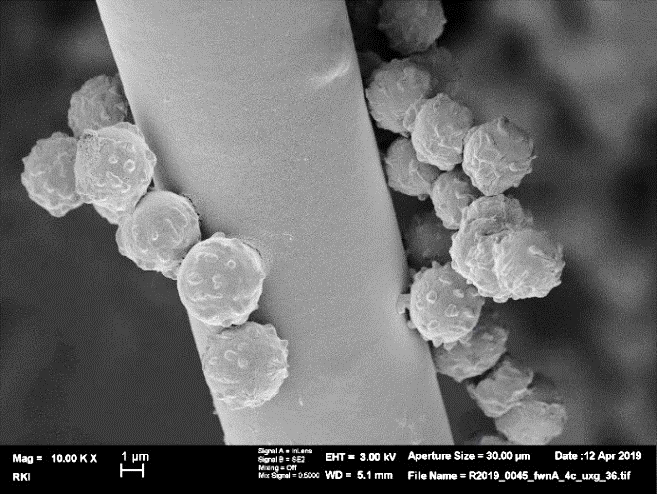

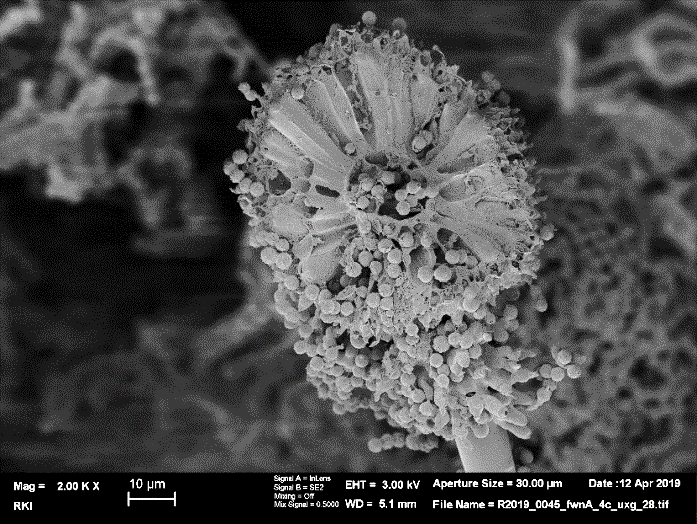

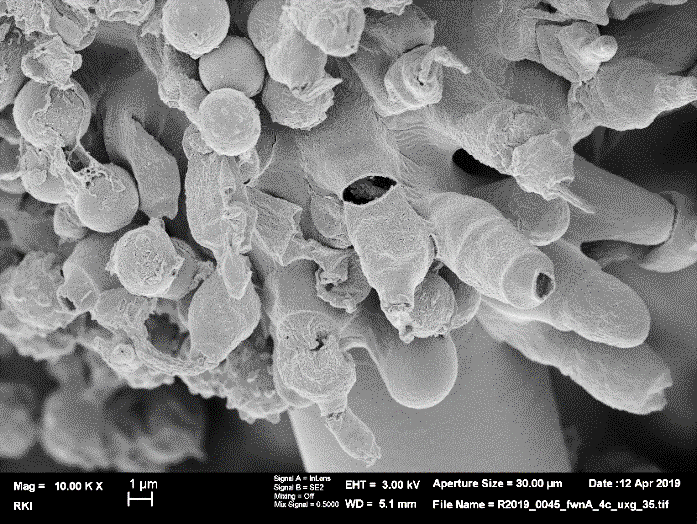


Hyperbranching mutant (∆*racA*)


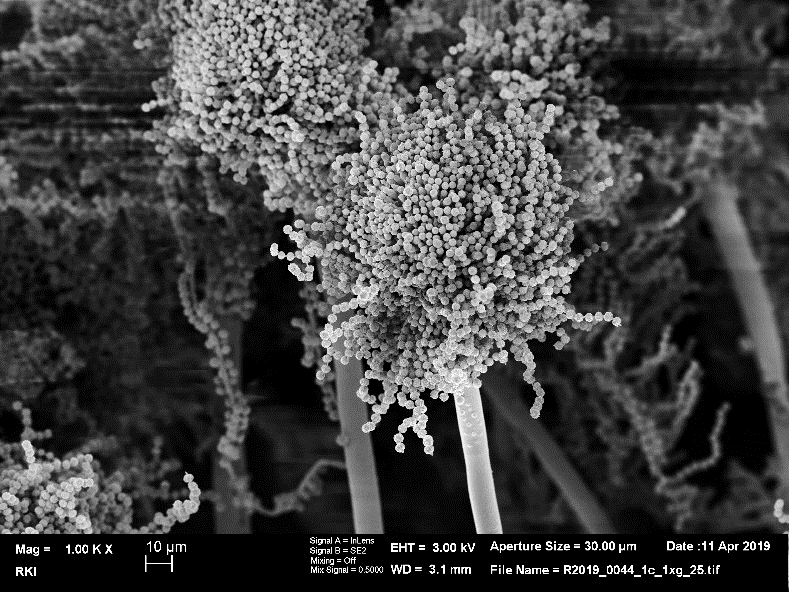


**10 µm**

**10 µm**

**10 µm**

**10 µm**

**1 µm**

**1 µm**

**1 µm**

**Supplemental Figure 1.** Conidiophore morphologies of *A. niger* strains. Arrows indicate irregular spore formation, through the conidiophore stalk (rather than through the vesicle) which occurred seldomly in the wild-type and Δ*fwnA* mutant but frequently in the Δ*racA* mutant.

Ground

SMG


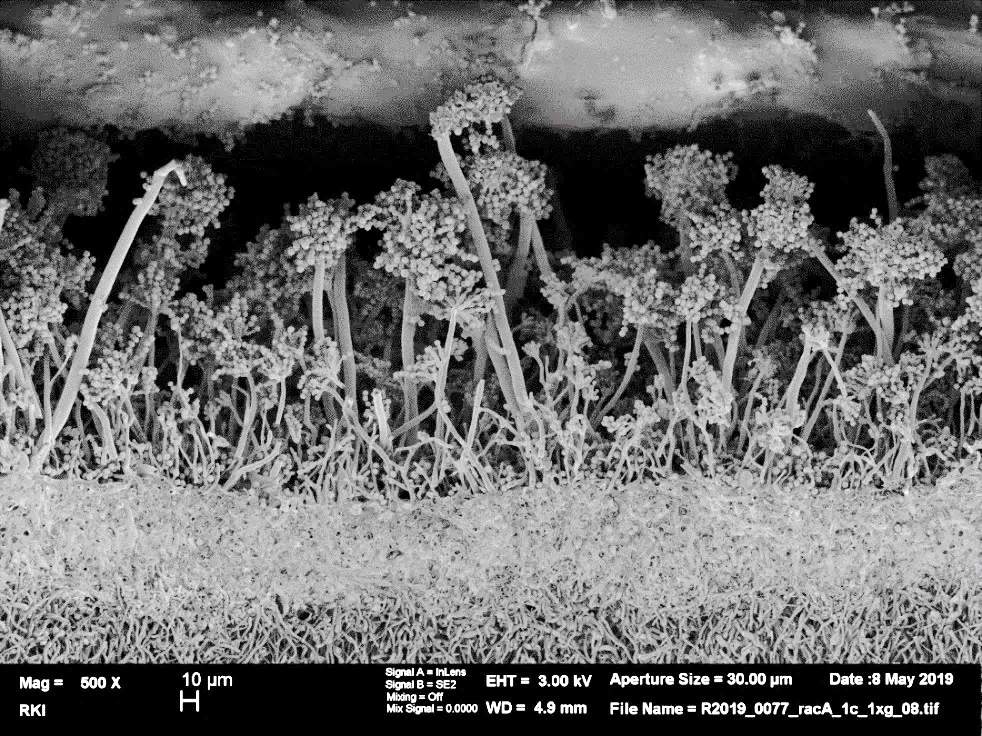

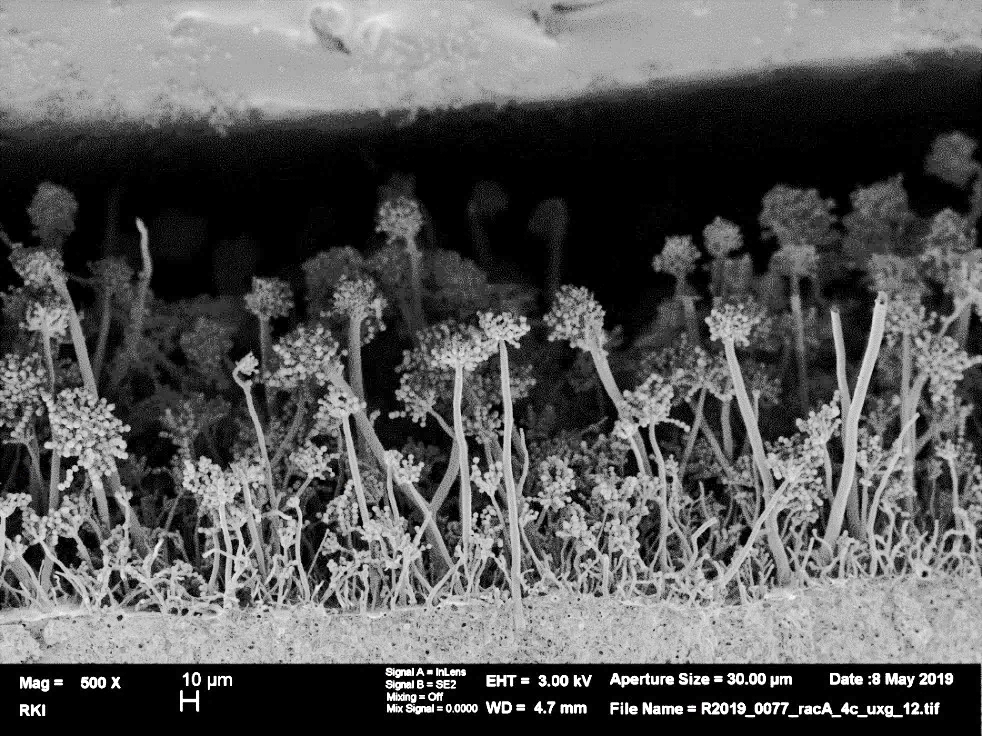


**10 µm**


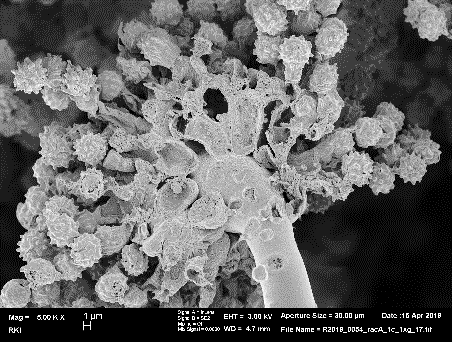

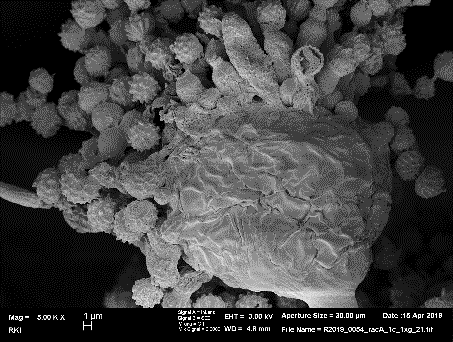

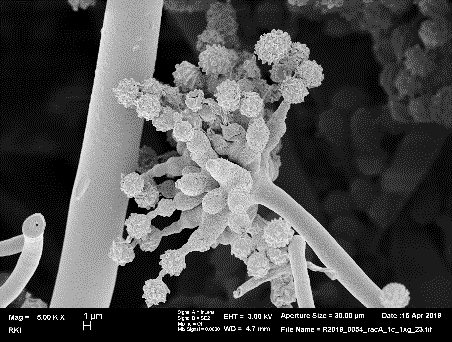

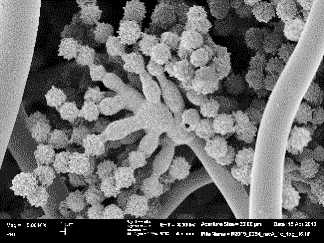


**1 µm**


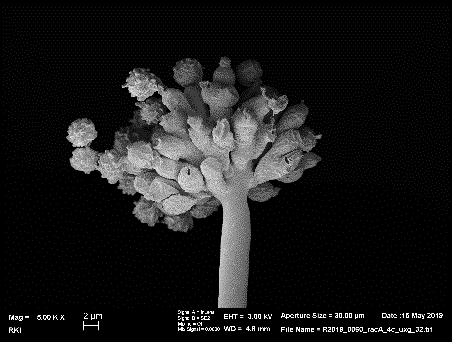

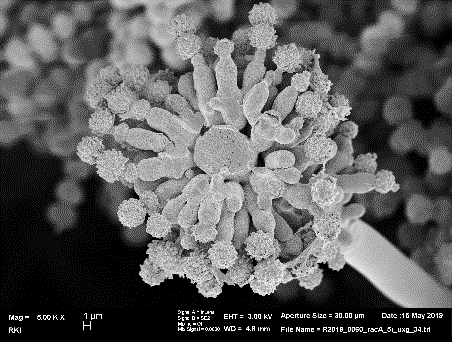

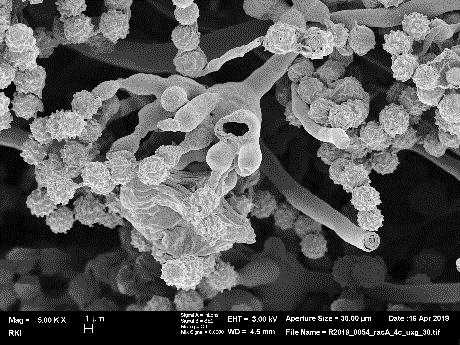

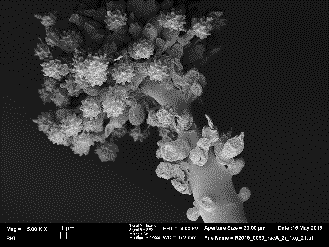


**8**

**5**

**1**

**2**

**4**

**7**

**3**

**6**

**b**

**a**

**10 µm**

**1 µm**

**1 µm**

**1 µm**

**1 µm**

**1 µm**

**1 µm**

**1 µm**

**Supplemental Figure 2.** Irregular conidiophore morphologies in the hyperbranching mutant strain ∆r*acA*. **a** Aerial mycelium showing conidiophores at different heights and morphologies. **b** Irregular vesicle formation: no vesicle (1-4), doubled vesicle (5) and vesicle with a divergent width (white line, 6-8). Irregularities were observed regardless of the gravitational regime.


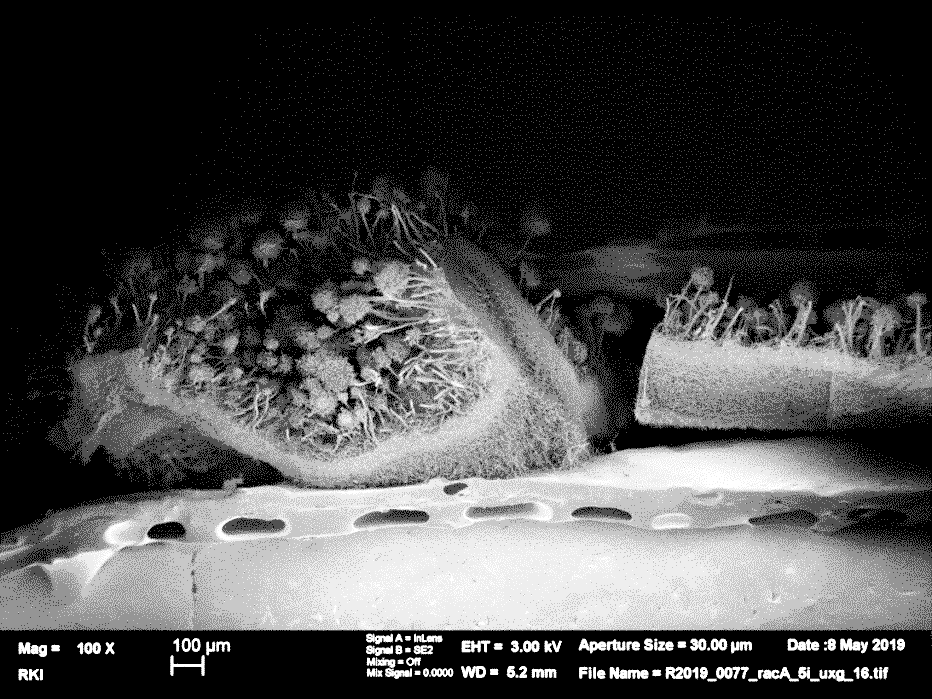

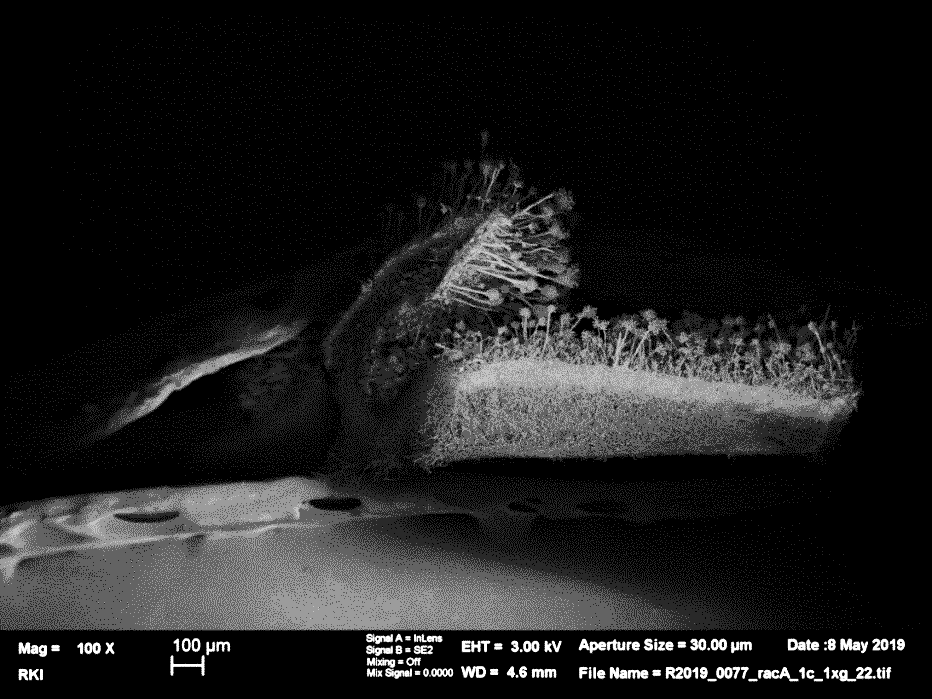

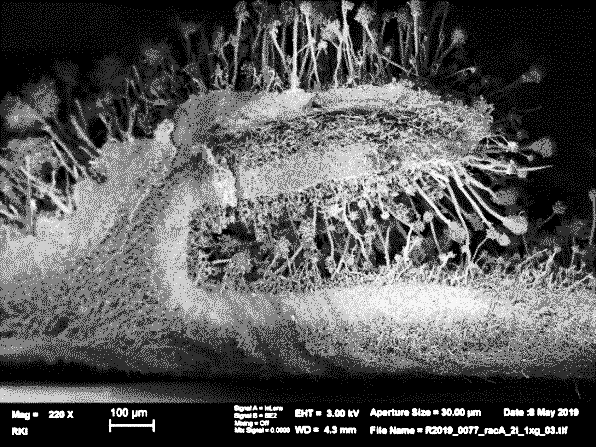

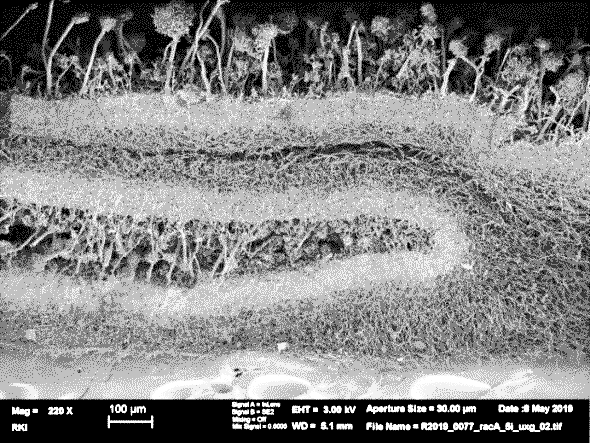


Ground

SMG


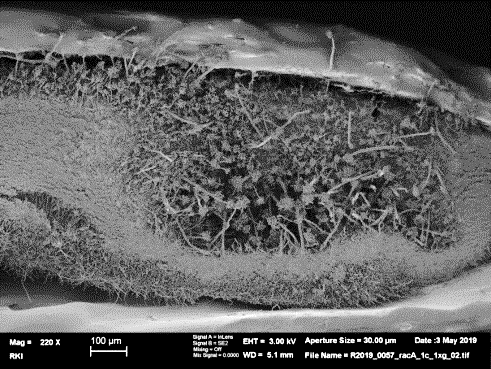

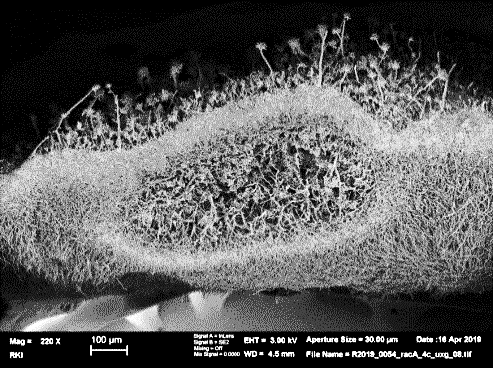


**100 µm**

**1**

**4**

**2**

**5**

**3**

**6**

**100 µm**

**100 µm**

**100 µm**

**100 µm**

**100 µm**

**Supplemental Figure 3.** Irregular colony morphologies in the hyperbranching mutant strain ∆r*acA*. Representative pictures of colony cross-sections show mycelium folds in both Ground (1-3) and simulated gravity conditions (4-6) after 5 days of cultivation on minimal medium agar plates. Note that aerial hyphae and conidiophores are formed in both directions: upward and downward. Marked squares show folded regions of the mycelium.
